# Revisiting Current Photoactive Materials for Antimicrobial Photodynamic Therapy

**DOI:** 10.3390/molecules23102424

**Published:** 2018-09-21

**Authors:** Mariana Q. Mesquita, Cristina J. Dias, Maria G. P. M. S. Neves, Adelaide Almeida, M. Amparo F. Faustino

**Affiliations:** 1 Department of Chemistry and QOPNA, University of Aveiro, 3810-193 Aveiro, Portugal; marianamesquita@ua.pt (M.Q.M.); cristina.jesus.dias@ua.pt (C.J.D.); 2 Department of Biomedical Sciences and iBiMED, University of Aveiro, 3810-193 Aveiro, Portugal; 3 Department of Biology CESAM, University of Aveiro, 3810-193 Aveiro, Portugal

**Keywords:** antimicrobial photodynamic therapy, photosensitizers, drug delivery, nanoparticles, silica, chitosan, cellulose, liposomes, hydrogels, nanotubes

## Abstract

Microbial infection is a severe concern, requiring the use of significant amounts of antimicrobials/biocides, not only in the hospital setting, but also in other environments. The increasing use of antimicrobial drugs and the rapid adaptability of microorganisms to these agents, have contributed to a sharp increase of antimicrobial resistance. It is obvious that the development of new strategies to combat planktonic and biofilm-embedded microorganisms is required. Photodynamic inactivation (PDI) is being recognized as an effective method to inactivate a broad spectrum of microorganisms, including those resistant to conventional antimicrobials. In the last few years, the development and biological assessment of new photosensitizers for PDI were accompanied by their immobilization in different supports having in mind the extension of the photodynamic principle to new applications, such as the disinfection of blood, water, and surfaces. In this review, we intended to cover a significant amount of recent work considering a diversity of photosensitizers and supports to achieve an effective photoinactivation. Special attention is devoted to the chemistry behind the preparation of the photomaterials by recurring to extensive examples, illustrating the design strategies. Additionally, we highlighted the biological challenges of each formulation expecting that the compiled information could motivate the development of other effective photoactive materials.

## 1. Introduction

Nowadays bacterial infections are still considered a severe burden worldwide, an issue that unfortunately tends to increase, not only in the clinic area, but also in other areas. This type of infection is one of the most serious causes of mortality of patients with traumatic lesions or after being subjected to surgeries, and also of animals, namely farmed ones [1,2]. Microorganisms replicate very fast and a mutation that benefits a microbe survival in the presence of an antimicrobial drug will rapidly become prevalent throughout the population. The inappropriate prescription of antimicrobials or their overuse exacerbates the problem [3]. The formation of biofilms is also responsible for an increase in the level of microbial resistance [4]. In fact, several microorganisms, such as bacteria, fungi, green algae, cyanobacteria, and lichen, possess the ability to grow in biofilm form and they are usually incorporated in a matrix of extracellular polymeric substances (EPS) auto-produced by the microorganisms, which makes the penetration of antimicrobial drugs difficult [5,6]. The organization in biofilms makes an organism more resilient to various environmental conditions and allows for its growth, even in hostile places [6,7,8]. The growth of biofilms in clinical settings, on buildings, monuments, and in technical installations, causes important health concerns as well as aesthetic, ecological, and economical damage. Additionally, spore-forming microorganisms, like bacteria and filamentous fungi, can cause severe problems to human health and their eradication is harder to achieve than in planktonic form [9,10]. Thus, significant amounts of antimicrobials/biocides are being used to combat planktonic and biofilm-embedded microorganisms’ growth in physiological fluids, waters, and on solid surfaces. However, the release of antimicrobials/biocides into the environment may induce new hazards to human health and environmental problems [11]. The adaptability of microorganisms caused by their fast reproduction rate is responsible for an increase in their resistance to the antimicrobials/biocides [6]. Therefore, it is of general consensus that the development of new strategies to combat planktonic, as well as biofilm-embedded microorganisms, is required.

Photodynamic inactivation (PDI) is an antimicrobial strategy that has been reported as an effective method to inactivate a broad spectrum of pathogens [12,13,14], including microorganisms that are highly resistant to conventional antimicrobials [15] and those that form biofilms [4,16,17,18].

**Figure 1:**
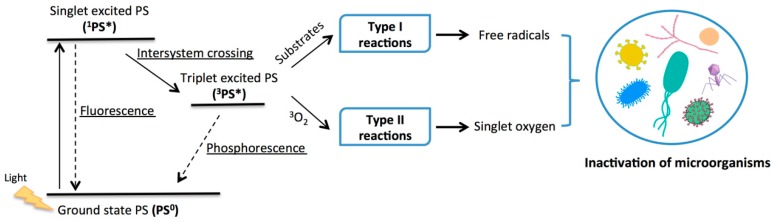
Illustration of the photochemical mechanisms of different reactive oxygen species (ROS) produced during photodynamic action.

This methodology requires the activation of a dye called a photosensitizer (PS) by an adequate light wavelength in the presence of molecular oxygen (Figure 1). The activated dye (PS*) then transfers its excess of energy or electrons to molecular oxygen (O_2_) or substrate molecules, generating highly cytotoxic reactive oxygen species (ROS), especially singlet oxygen (^1^O_2_) that oxidize different biomolecules (multi-target action) of planktonic or biofilm-associated microorganisms [19,20]. The lipids and proteins of the external structures of the microorganisms, cytoplasmic membrane, cell walls, capsids, and lipidic envelopes, are considered to be the main targets in PDI [21,22]. This oxidative stress induced by PDI leads to irreparable damage of these vital cellular components, causing microbial inactivation, avoiding the development of photoresistant strains [21,22,23,24].

**Figure 2:**
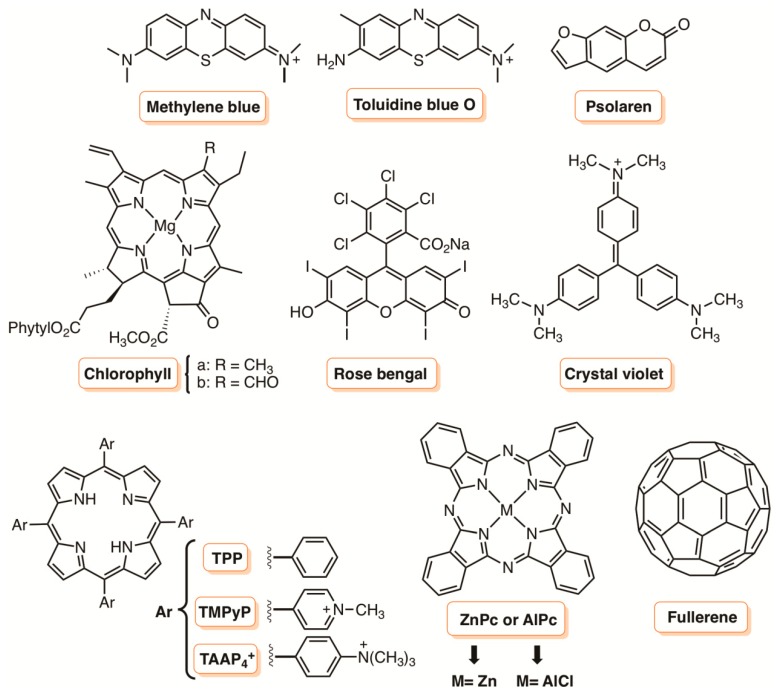
Structures of cores and some natural and synthetic PSs commonly used as antimicrobial agents.

**Figure 3:**
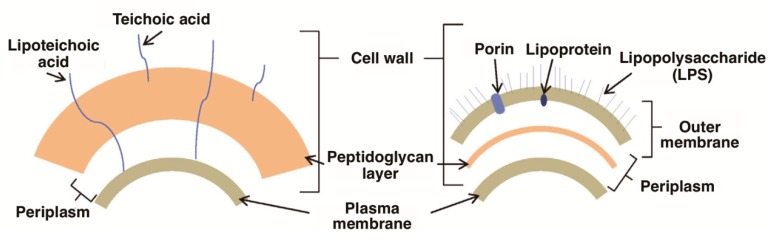
Schematic representation of the cellular envelope of Gram-(+) (**left**) and Gram-(−) bacteria (**right**). Reproduced from Reference [21] with permission of Elsevier.

In the last few years, the development and biological assessment of new molecules for PDI has grown considerably. Some classes of PSs, such as phenothiazinium dyes (e.g., methylene blue and toluidine blue O), psoralens, perylenequinonoid pigments (e.g., hypocrellin), natural and synthetic based tetrapyrrolic macrocycles (e.g., chlorophylls, bacteriochlorophylls, porphyrins, corroles, and phthalocyanines), and fullerenes have been effectively tested, demonstrating themselves to be good antimicrobial agents (Figure 2) [25,26,27,28,29,30,31]. For instance, the studies showed that the photoinactivation of Gram-(+) and Gram-(−) bacterial strains is highly dependent on the PS structure. In general, neutral, cationic, or anionic PS can easily inactivate Gram-(+) bacteria, but an effective photoinactivation of Gram-(−) bacteria, without the presence of membrane disrupting agents, requires the presence of a cationic PS. This inactivation difference is associated with the cell wall constitution. Most Gram-(+) bacteria have a single cell wall constituted by peptidoglycan, with lipoteichoic and teichuronic acids, which display a relatively high degree of porosity, while the Gram-(−) bacteria, other than the peptidoglycan layer, possess an additional highly organized outer membrane composed of lipopolysaccharides that are strongly negatively charged, phospholipids, lipoproteins, and proteins [31] (Figure 3).

The possibility of using the photodynamic approach to improve the microbiological quality of water and food, to control insect pests, and to disinfect and sterilize materials and surfaces in different contexts (industrial, household, and hospital) has been the research subject of several groups [23,32,33,34]. Additionally, the use of this approach to inactivate microorganisms has become increasingly achievable in practice if the PS is immobilized on inert solid supports [18]. Consequently, this will allow for the reuse and the materials recycling, making this methodology economic, sustainable, durable, and environmentally friendly [23,35,36]. Thus, the selection of the solid supports must be based on a few items: (i) compatibility with the PS, (ii) stability and mechanical strength towards light, (iii) easy and reproducible immobilization procedures; (iv) good oxygen permeability for efficient ^1^O_2_ production with minimum quenching, (v) commercial availability and low-cost, and (vi) high biocompatibility to maximize the interaction between the support and the microorganisms [37]. To this extent, some different supports have been created to immobilize PSs. The importance of this subject is demonstrated by the number of works and reviews that are appearing in the last decade [38,39,40]. Herein, we decided to revisit several recent innovative formulations, molecular designs, and modifications that demonstrated effective PDI of microorganisms, giving a special attention to the design of the synthetic strategies behind the preparation of the photoactive materials. In order to facilitate the discussion, the review is organized considering the type of support, namely metal nanoparticles (NPs), silica, biopolymers (chitosan and cellulose), liposomes, nanogels, and carbon materials. In terms of PS, special attention is given to some of the most studied options such as tetrapyrrolic macrocycles derivatives, phenothiazinium dyes, and fullerenes. However, when pertinent, some older works and research with other PSs will also be mentioned.

## 2. Metal Nanoparticles

The versatility of metal nanoparticles (MNPs) is well recognized in different areas such as engineering, chemistry, physics, biology, and medicine [41,42,43]. Some of their biological applications are related with their potential in the PDT of cancer, as drug delivery systems [44] and also as antibacterial and antifungal agents [45]. In fact, due to their small size, MNPs can attach to the cell wall of the bacteria or fungi through self-assembly causing cell death [46]. Additionally, some metals, like silver or zinc, are known for their natural antibacterial activity [47,48,49]. However, the MNPs must have the adequate dimension in order to avoid agglomeration/aggregation phenomena, which is related with a significant reduction of the antimicrobial effect. The mechanism of MNP action has not been fully understood. However, numerous theories were proposed based on the damage of the microbial enzymes by the release of metal ions, modifications in the membrane integrity with penetration into the bacterial cytoplasm, and accumulation in the periplasmic space, or due to the direct action of ROS generated by the effect of MNPs [50]. In this section, we will give an update on the antimicrobial properties of different types of MNPs associated with PS dyes on different microbial strains.

### 2.1. Gold Nanoparticles

**Figure 4:**
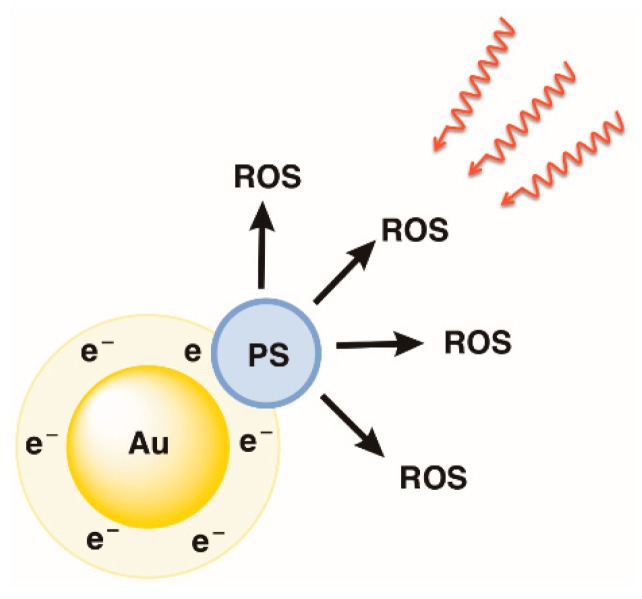
Plasmonic AuNP. The local electric field caused by conductance electrons potentiates the optical field near the surface and enhances the fluorescence or photoactivity of the attached PS (adapted from Reference [69]).

Among all the MNPs, gold NPs (AuNPs) have received particular attention due to their distinctive properties that can make them suitable for several applications such as labeling, delivery, heating, and sensing [51,52,53]. Recently, AuNPs also earned significant attention in the photodynamic approach due to their properties, such as, biocompatibility, size, unique surface, and also optical properties [54,55,56]. Furthermore, the conjugation of PSs on the surface of AuNPs may increase the PDI efficacy as a result of the localized surface plasmon resonance (SPR) of AuNPs upon light exposure. This situation will then lead to a PS-efficient activation (an increase in the PS excitation rate) and an improvement in the ROS production (Figure 4) [56,57].

In light of this, and considering that the colonization of catheters by microorganisms can be related with the development of some resistant biofilm-related infections, in 2009, Wilson and co-workers [58] prepared antimicrobial materials based on polysiloxane (a polymer used in catheters), methylene blue (MB; Figure 2), and 2 nm AuNPs and compared their photoefficacy with the polymeric matrix alone [58]. Small squares of the polymer were left for 24 h in the dark in solutions containing just MB (acetone–water solution) or MB and/or AuNPs. Then, the swelled polymers were removed from the solution and the solvents were allowed to evaporate in the dark for 24 h. The antimicrobial activity of prepared polymers was tested against the Gram-(−) *Escherichia coli* and the Gram-(+) methicillin-resistant *Staphylococcus aureus* (MRSA) and the results showed that the presence of AuNP potentiated approximately 2-fold the efficacy of MB. The polymer containing MB and AuNP showed 1.0 log reduction of *E. coli* after 5 min of irradiation with a red light (660 nm) provided from a handheld light system operating at 250 mW, versus 0.5 log with the polymer just containing MB. Similarly, the best bacterial reductions were observed for MRSA in the presence of the polymer containing MB and AuNP (3.5 log reduction), whereas with the MB polymer, a reduction of just 1.0 log was attained. No phototoxicity was detected either with the polymer alone or with just AuNP embedded. The best performance of the polymer containing both additives (MB and AuNP) was explained by recognizing the possible formation of other ROS besides ^1^O_2_, potentiated by the presence of AuNP [58]. The good results impelled the authors to consider the use of MB and AuNP embedded in a polysiloxane polymer to reduce the formation of *Staphylococcus epidermidis* biofilms [59]. The study was performed under the same laser irradiation (660 nm), but different exposure times and light regimes were assessed. The results showed that the best PDI combination (a reduction in biofilm coverage greater than 50% when compared to untreated polysiloxane polymer) was attained when the light was applied for 10 min every 60 min for a total period of 6 h (total energy dose of 117.0 J cm^−2^). Other combinations, like short irradiation periods (5 min) but frequent (every 30 min), or longer irradiation periods (20 min) but infrequent (every 120 min), were much less efficient, ending up in 75% and 60%, respectively, of the surface covered by the biofilm compared to 100% coverage when no light was used. The mechanical properties of the photoactive material (e.g., elasticity) were not affected, confirming the possibility to be incorporated in catheters in order to reduce catheter acquired infections [58,59]. Since then, and following the same approach, this group prepared other photoactive materials based on polymers (polyurethane, silicone) and other materials used in catheters and in hospital touch surfaces (screen protectors for telephones and tablets, covers, keyboards, and hand dryers) embedded with PSs [MB, toluidine blue O (TBO), crystal violet] and AuNP against methicillin-resistant *S. aureus* (MRSA), *S. epidermidis*, *Saccharomyces cerevisiae*, *E. coli*, Bacteriophage MS2, the fungus-like organism *Pythium ultimum*, and the filamentous fungus *Botrytis cinereal*, and the results showed an effective inactivation of all microorganisms [60,61,62,63,64,65,66,67,68].

**Figure 5:**
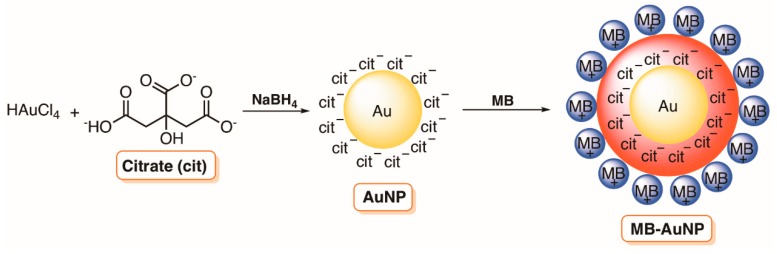
Schematic representation of the preparation of MB-AuNP conjugates (adapted from Reference [71]).

In 2015, Morsy and co-workers [70] also reported that the presence of 13 nm AuNP could improve the phototoxicity of MB towards an *S. aureus* strain isolated from childrens’ impetigo lesions; this strain is the most common organism found in impetigo infections and resistance to conventional antibiotics starts to be problematic. The authors prepared the PS-AuNP nanoconjugates by using the swell–encapsulation–shrink method. Succinctly, an aqueous solution containing colloidal AuNPs (obtained from a hot solution of HAuCl_4_ using trisodium citrate as a capping and reduction agent) and MB was maintained under stirring and ultra-sonication in order to improve the electrostatic interaction between both components. The MB-AuNP nanoconjugate prepared was assessed against *S. aureus* and compared with the photodynamic effect of AuNP and MB alone. As in the previous studies by Perni [58], the highest inhibitory effect on *S. aureus* was obtained with the conjugate MB-AuNP when irradiated for 2 min at 660 nm. Djavid and colleagues [71] extended the biological assessment of MB-AuNP nanoconjugate towards mature biofilms of MRSA. They prepared 5 nm MB-AuNP nanoconjugates and the successful conjugation between the positively charged MB dye and the citrate-stabilized AuNPs (Figure 5) at a molar ratio of 20/1 was confirmed by the presence of the characteristic AuNP absorbance peak at 520 nm in the UV-Vis spectrum.

The results showed that the MB-AuNP nanoconjugates were able to efficiently photoinactivate 1- and 4-day-old biofilms of MRSA after being exposed to light (650 nm) for 10 min (light irradiance of 22.93 J cm^−2^). In this case, a reduction higher than 5 log colony-forming unit (CFU) was attained, while with the non-conjugate MB, the reduction was smaller than 1 log CFU. This greater performance of the MB-AuNP nanoconjugate was attributed to two important features of AuNP: a high production of ROS due its localized surface plasmon resonance and to its role as a carrier allowed for delivering monomeric MB into the deeper part of the mature biofilm. The potential of the prepared conjugates to deal with chronic wound healing was also highlighted considering the minimal damage observed in the fibroblast cells [71].

### 2.2. Silver Nanoparticles

Silver NPs (AgNPs) are also being considered as an interesting alternative to antibiotic resistance due to powerful antimicrobial activity against both Gram-(+) and Gram-(−) bacteria of silver, accompanied by their low toxicity to mammalian cells at low concentrations [72,73,74,75]. Besides, AgNPs have also been proved to disrupt the biofilm formation [76]. In fact, the possibility of developing a new generation of antibiotics based on AgNPs merits special attention from the scientific community. Although, the mechanisms underlying the action of AgNPs towards microorganisms are not fully clarified, it is accepted that the possible development of resistance by microorganisms is unlikely due to the multitarget action of silver [41].

**Figure 6:**
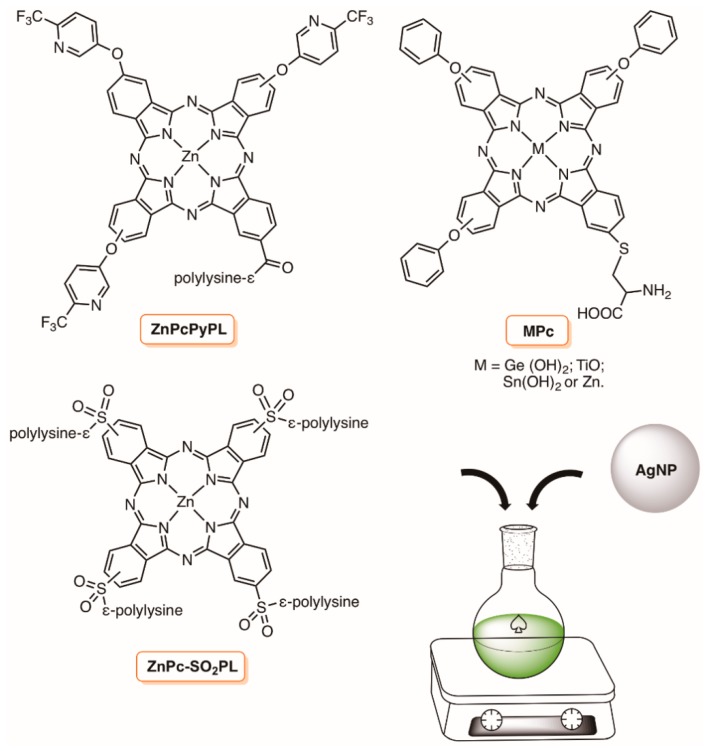
Structures of metal phthalocyanines used in the preparation of phthalocyanine-AgNPs conjugates (adapted from References [66,67]).

In 2012, Nyokong and co-workers reported that the conjugation of spherical 7 nm AgNP to the zinc(II) complex of phthalocyanines bearing poly-lysine chains (ZnPcPyPL and ZnPc-SO_2_PL) (Figure 6) significantly improved the *S. aureus* growth inhibition when compared to the results achieved with the corresponding phthalocyanines alone. The conjugation onto the AgNP surface was potentiated by the presence of amino groups in the ZnPc poly-lysine chains, and it was achieved by stirring both components at room temperature for 15 min (Figure 6) [77].

As an extension of the previous studies, the same group reported the synthetic access to a series of conjugates involving the non-symmetric metallophthalocyanines MPc bearing a cysteinyl unit (Figure 6) with different shapes of AgNPs (spherical, cubes and triangles) [78]. The presence of the –NH_2_ and –COOH groups on the phthalocyanine was important in the stabilization of the MPc-AgNP nanoconjugates. The antimicrobial activity of the prepared nanoconjugates was evaluated against *S. aureus* in the dark and after irradiation with visible light for 90 min. The highest antimicrobial activity found for the spherical AgNPs alone was explained by considering their smaller size (15 nm) and consequently more surface area when compared to triangles (size 54 nm) and cubes (60 nm) shaped AgNPs. The results also showed that the toxicity of the MPc derivatives was improved by irradiation and the photodynamic inactivation efficiency of all MPc-AgNPs nanoconjugates was slightly higher than that of MPcs alone, although following the same order (Sn < OTi < Zn < (OH)_2_Ge). The spherical MPc-AgNPs nanoconjugates also presented the best antimicrobial performance, and the high efficacy of the germanium phthalocyanine complex was associated to its high efficacy to generate ^1^O_2_, either alone or after AgNP conjugation [78].

Promising results concerning the combination of AgNPs with a PS towards Gram-(−) *Pseudomonas aeruginosa* were also reported by Lyutakov et al. [79]. The study involved the preparation of films based on poly(methylmethacrylate) (PMMA) doped with 5,10,15,20-tetraphenylporphyrin (TPP; Figure 2) and AgNPs [79]. These films (TPP-AgNPs/PMMA) were prepared by dissolving silver nitrate, TPP, *N*-methylpyrrolidone, and PMMA in dichloroethane. Then, after spin-coating at 1000 rpm for 15 s, the solvent was evaporated in a hot plate (200 °C). A similar approach was used to prepare PMMA films with just TPP or AgNPs for comparison. The antimicrobial results showed that the presence of both components was important for an effective reduction of Gram-(−) bacteria under blue (405 nm) LED light irradiation. The authors considered that the specific antimicrobial properties of AgNPs/PMMA films towards the Gram-(−) bacteria (through silver release) and the photoactivity of TPP/PMMA against *S. aureus* (through ROS formation) could justify the better performance of the antimicrobial material comprising both components [79].

**Figure 7:**
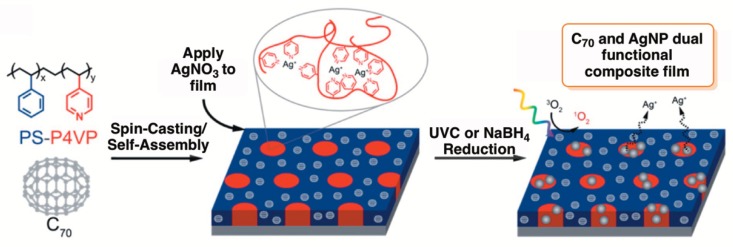
Schematic representation of the procedure to prepare PS-P4VP multifunctional antimicrobial films containing C_70_ and in situ prepared AgNPs. Reproduced from Reference [80] with permission from the American Chemical Society.

A similar approach was developed by Moor et al. [80] to create multifunctional antimicrobial materials, where two independent components (fullerenes and AgNP) were combined for a synergistic enhancement of antimicrobial character of thin films against *E. coli* and bacteriophage PR772. The authors used polystyrene-block-poly-4-vinylpyridine (PS-P4VP) copolymers with two domains to integrate fullerene molecules (C_60_ and C_70_) into the polystyrene domain and the in situ formed AgNP into P4VP domains (Figure 7). The results revealed that C_70_ loaded PS-P4VP films were able to produce an upper amount of ^1^O_2_ and displayed a significantly higher antimicrobial activity (against bacteriophage PR772 and *E. coli*) after light activation than the corresponding analogous C_70_ loaded homopolymer film (without AgNP incorporated), which confirmed the dual functionality of the produced film [80].

### 2.3. Platinum Nanoparticles

Considering the antibacterial features of platinum, the development of materials based on platinum NPs (PtNPs) associated to PS molecules also merited some attention from several research groups. Platinum is known to inactivate microbes by interacting with their enzymes, proteins, or DNA, and to restrain cell proliferation or cell division [81]. Thus, the combined action of PSs with PtNPs for photodynamic inactivation of microorganisms may cause a synergistic effect, resulting in improved antibacterial activity.

**Figure 8:**
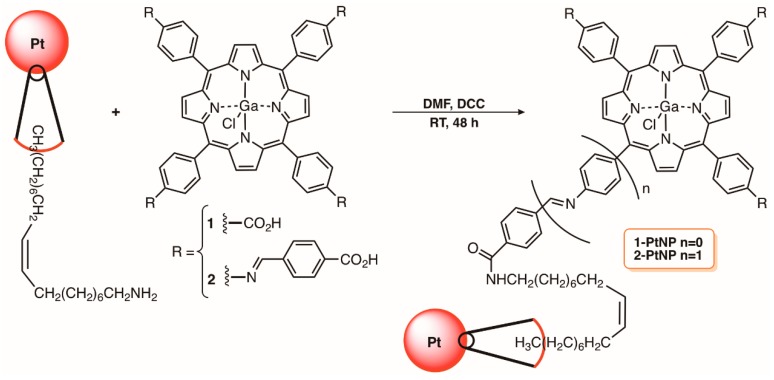
Synthetic access to conjugates involving acid porphyrins and PtNP (adapted from Reference [82]).

The studies of Nyokong and co-workers are in accordance with the beneficial effect of combining PtNPs with PSs [46,82]. The authors reported the conjugation of different shapes of PtNP (cubic, hexagonal, and unshaped) with 5,10,15,20-tetrakis(4-carboxyphenyl)porphyrinatogalium(III) chloride (1, ClGaTCPP) and evaluated their antimicrobial activity towards a drug-resistant Gram-(+) *S. aureus* strain in solution and embedded in polystyrene nanofibers (Figure 8). The PS units were covalently conjugated to the PtNP through an amide bond involving the oleylamine amine groups present on the PtNP surface and the carboxylic groups of the porphyrin (Figure 8). The different shaped conjugates 1-PtNPs were then suspended in a dimethylformamide/tetrahydrofuran (DMF/THF) (1:1) solution containing polystyrene polymer (1:10) and were electrospun into nanofibers with diameters ranging 6–14 µm. The studies showed a better antimicrobial activity of the conjugates than when the porphyrin ClGaTCPP was used alone and the higher reduction was attained when the porphyrin was conjugated to cubic PtNPs (4.64 log indicating that 99.99% of the bacteria have been killed); ClGaTCPP (1) alone caused a reduction of 3 log.

The same authors extended the strategy to another tetracarboxylic porphyrin derivative 2 (Figure 8), which was also covalently immobilized into PtNPs through an amide bond, and assessed its photoefficacy against *S. aureus*, *E. coli*, and *Candida albicans* in solution and after being embedded in polystyrene nanofibers [83]. Once again, an increased antimicrobial photodynamic effect was observed in the presence of the porphyrin conjugated 2-PtNP when compared with the porphyrin 2 and PtNP tested alone for all microorganisms [83].

## 3. Silica Supports

Among the nanocarriers used as drug delivery systems, silica has been extensively studied due to its biocompatibility, versatility, and easy preparation under mild conditions from readily available precursors [84,85]. The approaches usually used to immobilize the PS in this type of support can involve adsorption, entrapment during inorganic matrix preparation, or covalent bonding to the matrix via surface OH or other present groups.

Some of the prepared materials appeared as an opportunity for water treatment purposes [86,87,88]. In fact, materials based on non-magnetic and magnetic silica NPs with different PSs covalently immobilized have been recently developed, in order to facilitate their removal from the water matrix, for subsequent reuse [23]. Additionally, silica NPs loaded with antimicrobial agents have been shown capable to revert antibiotic resistance [84,89,90,91]. In particular, the immobilization of a PS on a silicate matrix possesses some benefits when compared with organic matrices since it is insoluble in water, resistant towards microorganisms, easy to fabricate, and can be successfully developed for the photo-disinfection of water or physiological fluids [35,92].

**Figure 9:**
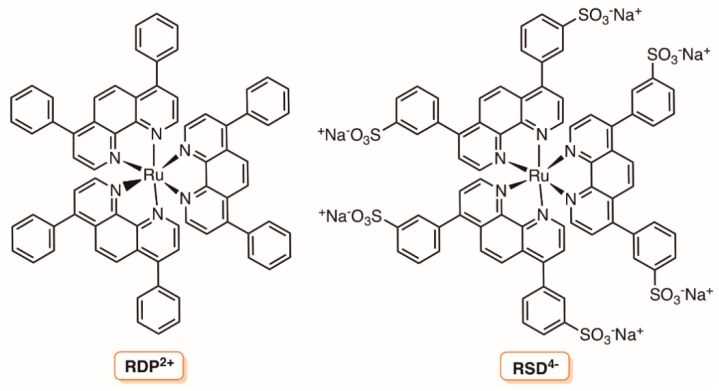
Structure of the ruthenium(II) complexes RDP^2+^ and RSD^4−^ with polyazaheterocyclic (adapted from Reference [96]).

Under the context of water disinfection, Orellana and Fresnadillo and their co-workers developed materials based on Ru(II) complexes immobilized in silicone [37,93,94,95,96,97]. In one of their studies [96] the authors reported the synthetic access to photosensitizing materials based on the ruthenium complexes tris(4,7-diphenyl-1,10-phenanthroline)ruthenium(II) dichloride, (RDP^2+^) and tetrasodium tris(1,10-phenanthrolinyl-4,7-bis(benzenesulfonate)ruthenate(II) (RSD^4−^) (Figure 9) electrostatically immobilized, respectively, on commercially available anionic and cationic porous silicone (pSil^−^ and pSil^+^). Then, the authors evaluated the efficiency of prepared materials against a Gram-(+) bacterium *Enterococcus faecalis*. Several photosensitizing materials based on the RDP^2+^ complex and pSil^−^ were prepared in the load range of 0.75–4.40 g m^−2^ without achieving surface saturation, but with the anionic RSD^4−^ sensitizer, due to its solubility in polar solvents, and the load on pSil^+^ was limited to 0.03 g m^−2^. The photodisinfection assays performed under solar-simulated radiation (20 W m^−2^) showed that the best performance was obtained with the RDP^2+^/pSil^−^ material with the highest sensitizer load (4.40 g m^−2^), reaching a bacterial reduction of 2 orders of magnitude; under sunlight (ca. 400 W m^−2^) the same material was able to provide a full disinfection after 45 min of irradiation while a decrease of 50% was observed with the support pSil^−^ alone. The antibacterial efficiency differences observed for the two prepared materials (RDP^2+^/pSil^−^ and RSD^−^/pSil^+^) under solar-simulated radiation was explained by the ^1^O_2_ production differences, which is probably related to the limited load of the RSD^4−^ PS. Thus, it was concluded that the ^1^O_2_ generation and the stability of the material, and consequently the bacteria photoinactivation efficiency, were related with the sensitizer charge, its load, and the ionic character of the porous silicone.

Under the same context of water disinfection, the use of silica magnetic nanoparticles as a template to disperse the PS also deserved some attention from the scientific community considering the photobactericidal material is easily recovered just by applying a magnetic field [35,98,99].

**Figure 10:**
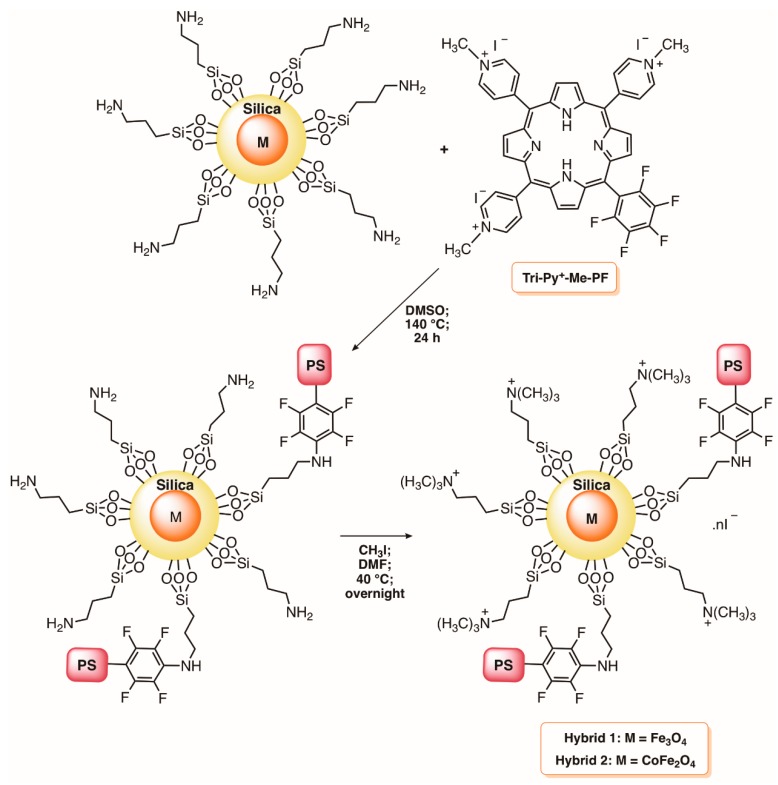
Preparation of silica nanomagnet-porphyrin hybrids 1 and 2 (adapted from References [35,99]).

In 2010, considering the remarkable photodynamic efficiency of 5,10,15-tris(1-methylpyridinium-4-yl)-20-(pentafluorophenyl)porphyrin tri-iodide (Tri-Py^+^-Me-PF) towards a wide range of microorganisms under mild conditions [100], our group reported its immobilization in cationized silica-coated magnetic nanoparticles of Fe_3_O_4_ (Figure 10) [35]. The multi-charged nanomagnet Hybrid 1, based on Tri-Py^+^-Me-PF, was effective in the photoinactivation of both Gram-(+) and Gram-(−) fecal bacteria, causing a 5 log decrease for *E. faecalis* and *E. coli* at 20 μM of Tri-Py^+^-Me-PF when using white light irradiation (64.8 J cm^−2^). The full inactivation of the T4-like bacteriophage (≈7 log of inactivation) in the presence of the same hybrid also confirmed its efficacy as an antiviral agent [35]. The capability of this hybrid material to be recycled and reused, and also the potential of the analogue with a CoFe_2_O_4_ core (Hybrid 2), were posteriorly evaluated using water contaminated with the Gram-(−) bacterium *Allivibrio fischeri* (Figure 10) [99]. The results obtained demonstrate that both hybrids were effective in the bacterial inactivation with accumulative values after a six-cycle reuse of ≈42 log CFU mL^−1^ in 21.5 h and ≈38 log CFU mL^−1^ in 27 h, respectively, for Hybrids 1 and 2. It was commented that the efficacy of the recycling process could be improved if bigger active particles were used to be completely retained by a magnetic field [99].

**Figure 11:**
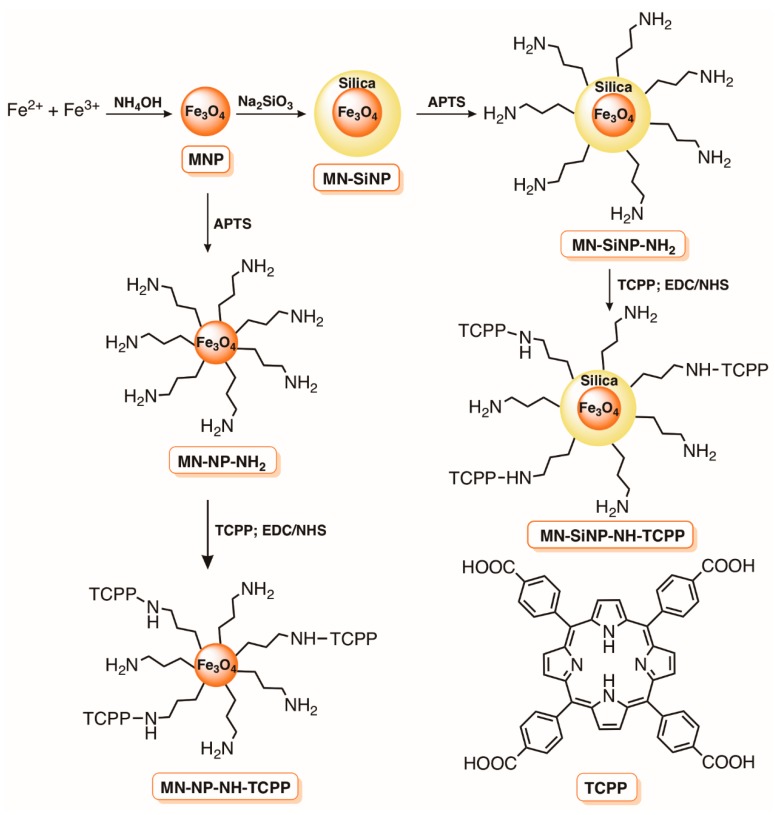
Representation of the synthetic access to nanomagnet materials MN-SiNP-NH-TCPP and MN-NP-NH-TCPP (adapted from Reference [98]).

Durantini and co-workers [98] also selected magnetic SiNP of Fe_3_O_4_ functionalized with aminopropyl groups to covalently link the 5,10,15,20-tetrakis(4-carboxyphenyl)porphyrin (TCPP) (Figure 11) in order to evaluate their potential as an antimicrobial material. In the material referred to as MN-SiNP-NH-TCPP, the magnetic core was previously coated with silica before functionalization with (3-aminopropyl)trimethoxysilane (APTS), while in the other material, designed as MN-NP-NH-TCPP, the grafting of the amino functionalities was performed directly on the magnetic core. In both cases, the conjugation of porphyrin derivative in the magnetic NP was successful attained via carbodiimide activation of the carboxylic groups (Figure 11). The biological studies revealed that both materials were effective against *S. aureus*, *E. coli*, and *C. albicans* in the first cycle of inactivation but the efficiency of MN-NP-NH-TCPP rapidly decreased in further cycles. A different situation was observed with the magnetic coated material MN-SiNP-NH-TCPP; the efficient decrease of 2.5 log for *S. aureus* and *C. albicans* and 3.0 log for *E. coli* after 30 min of irradiation (90 mW cm^−2^) at 3.0 µM of TCPP observed in the first inactivation cycle was maintained in the following two cycles. In this study, it was proved that ^1^O_2_ was the main cytotoxic species involved in the photodynamic process and the presence of the silica coating layer in the MN-SiNP-NH-TCPP was important to avoid the magnetic core oxidation and decomposition, and to prevent aggregation [98].

**Figure 12:**
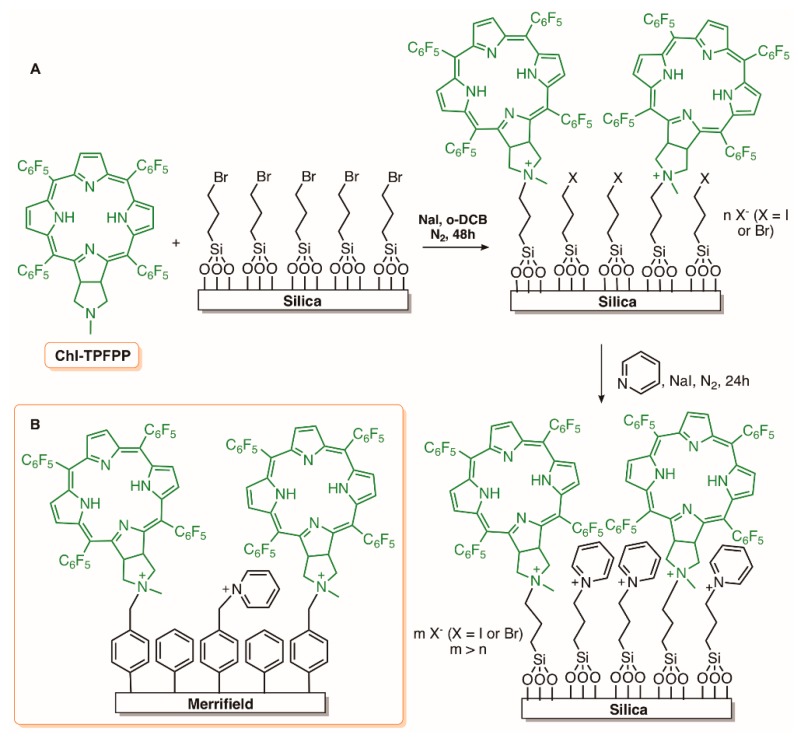
(**A**) Schematic representation of the immobilization of a pyrrolidine-fused chlorin derivative on a modified silica gel support with further cationization with pyridine units. (**B**) Diagram of the pyrrolidine-fused chlorin derivative immobilized on a cationized Merrifield resin (adapted from Reference [101]).

Considering the suitability of the pyrrolidine-fused chlorin derivative (Chl-TPFPP) obtained from 5,10,15,20-tetrakis(pentafluorophenyl)porphyrin) (TPFPP) to act as nucleophile, our group envisaged its immobilization on the commercial available 3-bromopropyl-functionalized silica and also to Merrifield resin-based materials, as an economically viable and environmental friendly approach to allow successive recovery and removal of the PS material after photodynamic treatment (Figure 12A,B) [92,101]. The study indicated that the materials resulting from the immobilization of the chlorin derivative on both commercial materials followed by further treatment with pyridine had high potential as PSs for the inactivation of *E. coli* (*ca* 3.0 log reductions) with an irradiance of 4.0 mW cm^−2^ after 180 min. Additionally, both materials (silica and Merrifield resin based) showed good photostability under PAR white light (380–700 nm) and after three successive cycles of bacterial photoinactivation, the bacterial reduction was kept constant. So, the prepared photoactive materials can be considered as an inexpensive and friendly option for application in clinic and environmental areas [85].

**Figure 13:**
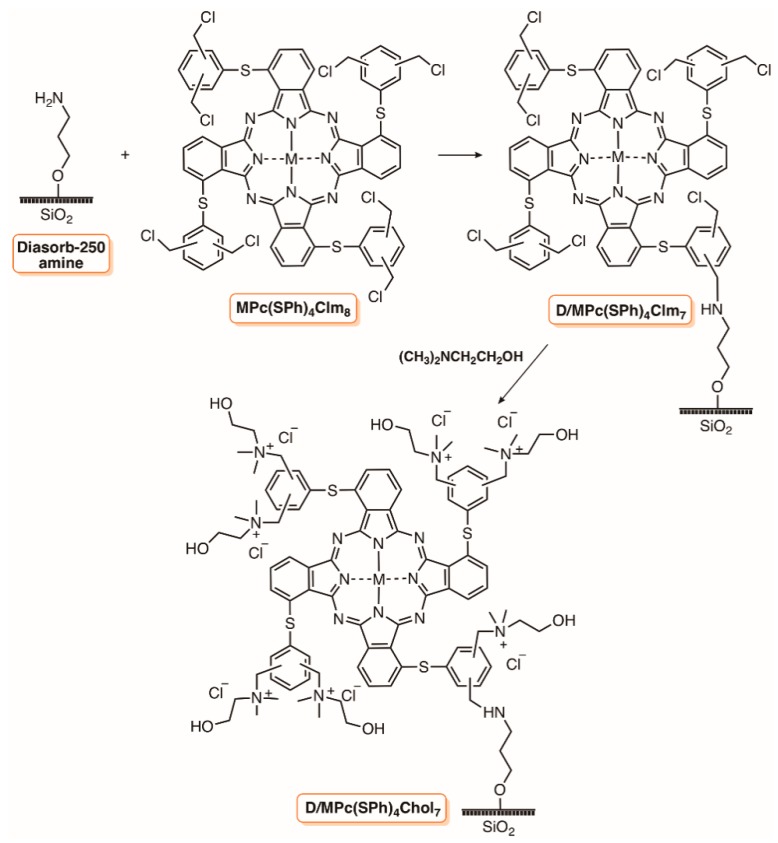
Synthetic route for the preparation of the metallophthalocyanines supported on silica materials (adapted from Reference [102]).

Kuznetsova et al. [102] selected the commercially available aminopropyl silica to immobilize the zinc and aluminum complexes of phthalocyanines tetrasubstituted at non-peripheral positions with chloromethylated thiophenyl groups as potential photobactericidal materials (Figure 13). The grafting of the phthalocyanines in silica was performed in DMF at ≈85 °C and further reaction with *N*,*N*-dimethylaminoethanol gave rise to positively charged D/MPc(SPh)_4_Chol_7_. Sodium taurinate was selected to obtain negatively charged phthalocyanines. The photodynamic efficiency of the different materials was evaluated using a bioluminescent *E. coli* and the biological assays were performed under white light (75 mW cm^−3^) at a PS concentration of 20 µM. The results showed that the aluminum positively charged phthalocyanine D/AlPc(SPh)_4_Chol_7_ had the highest bacterial activity (bioluminescence reduction > 80%), followed by neutral D/AlPc(SPh)_4_Clm_7_ (80%), and finally by the negatively charged taurinate phthalocyanines (bioluminescence reduction ≈60%). The authors commented that a more effective interaction between the Gram-(−) bacteria and the positively charged PS was probably responsible for this fact. The dependence observed on the cationic D/ZnPc(SPh)_4_Chol_7_ to generate ^1^O_2_ with the load in the support (less PS load means better efficiency) was not reflected in its photodynamic action, since a similar bioluminescence reduction of ≈70% was found for materials with different zinc(II) phthalocyanine loads [102].

**Figure 14:**
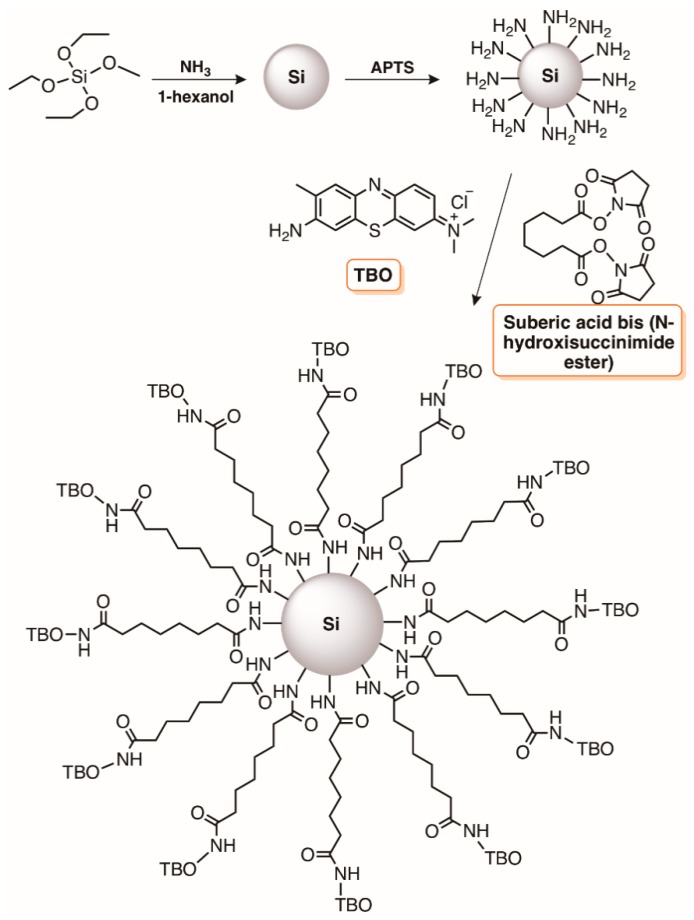
Structure of Toluidine Blue O silica nanoparticle (adapted from Reference [103]).

Light-activated silica NPs adequately functionalized with acids groups were used as templates to immobilize Toluidine Blue O (Figure 14) giving rise to materials with promising photobactericidal activity against *S. epidermidis*, *E. coli*, and MRSA [103]. After irradiating the samples with a light of 630 nm, delivered by a 500 mW laser, a bacterial reduction of 2 log was observed for *S. epidermidis* and *E. coli* after 2 and 3 min of treatment, respectively. Also, the number of viable MRSA cells after laser irradiation in the presence of the SiNP-TBO nanoconjugates decreased with the increase of the treatment time. The higher difficulty to photoinactivate *E. coli* when compared to *S. epidermidis* and MRSA was explained by considering the more complex cell wall structure of Gram-(−) bacteria when compared with Gram-(+). Moreover, no antimicrobial activity was observed by the unconjugated silica or by the laser light alone. With these results, the authors concluded that the conjugates could be used to prepare materials, such as hydrogels and topical creams, that exhibit light-dependent antimicrobial activity [103].

**Figure 15:**
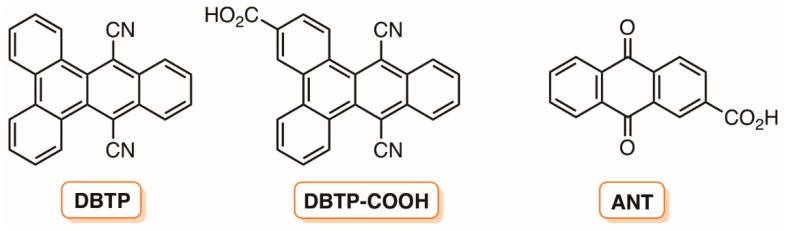
Structures of the two different PSs covalently linked to silica [104].

Lacombe and their co-workers [104] also reported the synthesis and the disinfection efficiency towards *E. coli* of silica-based materials covalently linked to other non-porphyrinic PSs as 9,10-anthraquinone-4-carboxylic acid (ANT) and 9,14-dicyanobenzo[*b*]triphenylene-3-carboxylic acid (DBTP-COOH) (Figure 15). The immobilization of ANT involved a preliminary functionalization with (3-aminopropyl)triethoxysilane followed by the grafting in commercial silica beads by reflux in toluene affording SiO_2_-ANT. The DBTP was grafted directly in a commercially available amino-functionalized silica powder giving rise to the material designed as SiNH_2_-DBTP. Although a slow but effective inactivation of *E. coli* (7 log after 360 min of irradiation) was observed with pure silica (without any PS) under UV-A (3.85 mW cm^−2^, 365 nm), a noticeably improvement was detected when the irradiation took place in the presence of the same amount (2.5 g L^−1^) of SiO_2_-ANT and to a lesser extent with SiNH_2_-DBTP at the same concentration. After exposure to UV-A for 110 min and 270 min, respectively, SiO_2_-ANT and SiNH_2_-DBTP were able to decrease the bacterial concentration to undetectable levels (decreases of > 6.0 log). It was also demonstrated that the mode of PS introduction in silica and the amount of silica in the suspension were fundamental parameters for the material efficiency [104].

**Figure 16:**
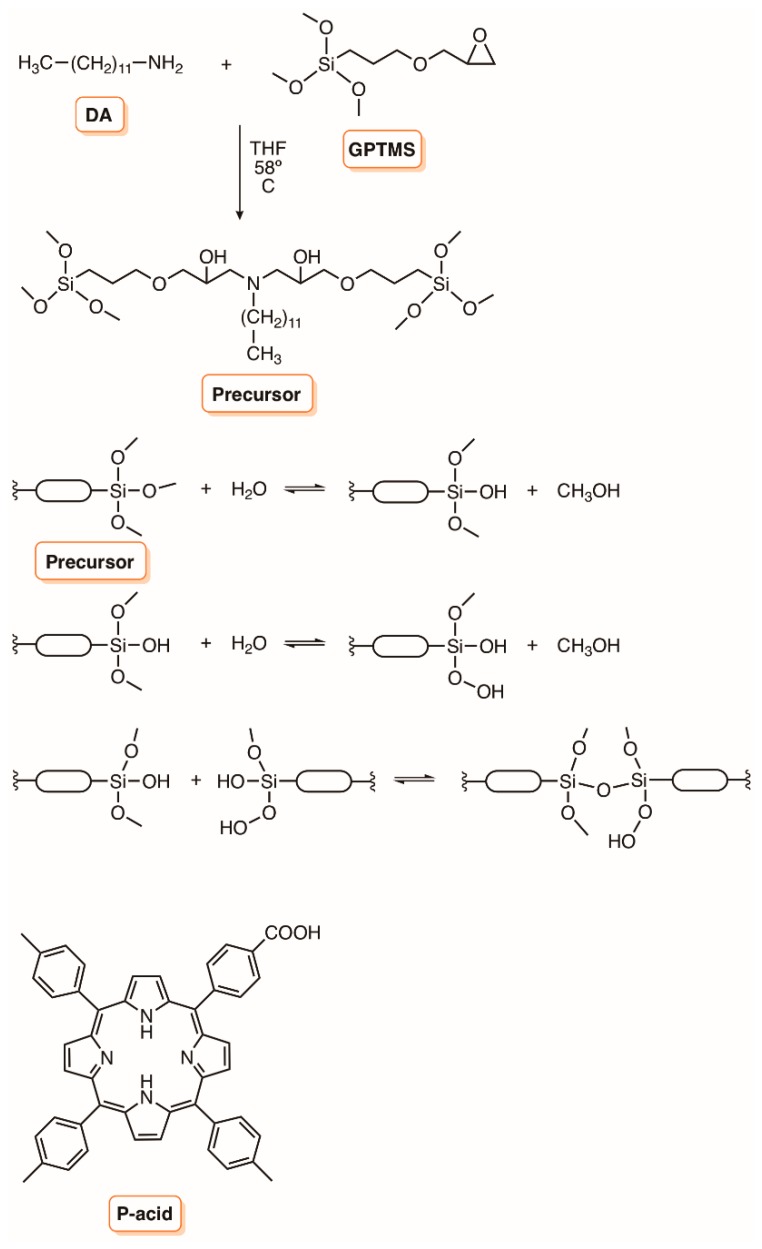
Preparation of the polysilsesquioxane flexible films doped with 5-(4-carboxyphenyl)-10,15,20-tris(4-methylphenyl)porphyrin (adapted from Reference [105]).

The development of surfaces based on bridged polysilsesquioxane films doped with the non-symmetric porphyrin 5-(4-carboxyphenyl)-10,15,20-tris(4-methylphenyl)porphyrin (P-acid, Figure 16) is another approach, giving rise to materials with promising antifungal activity [105]. The precursor of silsesquioxane was prepared from a (glycidoxypropyl)trimethoxysilane (GPTMS) and dodecylamine (DA) reaction, and the incorporation of the porphyrin, predominantly as monomer, occurred during the acid hydrolysis of the trimethoxysilane groups [Si(OCH_3_)] and polycondensation reaction to form bridged polysilsesquioxane plastic films. The absence and the presence of the porphyrin in the flexible films was confirmed by the tonality acquired. The antifungal activity photoinduced by the prepared films was evaluated against *C. albicans* in aqueous suspensions and on its surface. It was observed a 99.7% of fungal inactivation after 60 min of irradiation (90 mW cm^−2^) with visible light. Also, they were effective in photoinactivating *C. albicans* dropped on the film surface, with a 95% decrease in cells survival after 30 min of irradiation. The results showed that the inactivation of *C. albicans* was mostly mediated by ^1^O_2_ [105].

## 4. Biopolymers

The design of new photodynamic antimicrobial materials based on non-toxic and biodegradable supports, such as chitosan, cellulose, and their derivatives, is also having some impact on the research concerning the recovery and reuse of PSs in PDI, as it is evident in the works reported in the following sections.

### 4.1. Chitosan

Chitosan is a poly(d-glucosamine) that is easily isolated from the chitin exoskeleton of crustacea using acid hydrolysis [106]. Chitosan displays attractive properties to be used as a PS support such as its film-forming ability, biodegradability, and an inherent antimicrobial activity related with the protonated amino groups. The antimicrobial activity of this polymer can be improved through the covalent or non-covalent incorporation of bioactive materials/compounds like PS, phenolic compounds, antibiotics, and tetrahydrocurcominoid derivatives [30,106,107,108,109,110,111].

**Figure 17:**
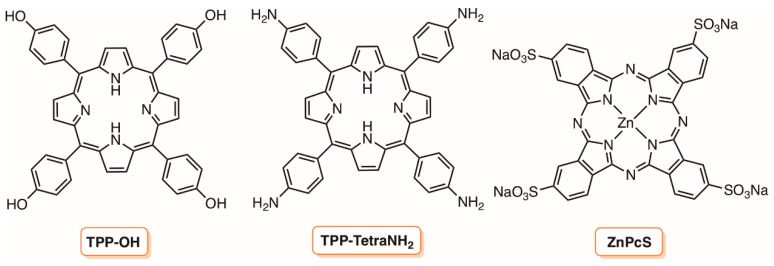
Structures of the porphyrins and phthalocyanine derivatives used by Bonnett and co-workers (adapted from Reference [106]).

One of the first references where chitosan was recognized to have the potential to immobilize PSs for the photodynamic inactivation approach was reported by Artarsky and co-workers in 2006 [106]. The authors selected chitosan membranes (simple or reinforced in nylon) to immobilize two porphyrins and a phthalocyanine (Figure 17) as a proof of concept that the photodynamic effect could be used to lower microbial levels in water under flow conditions. The authors considered that the hydrophilicity (wettable) of the polymer would facilitate the contact between the PS and the microorganisms and the incorporation of those PSs involved three different approaches. The 5,10,15,20-tetrakis(4-hydroxyphenyl)porphyrin (TPP-OH), was incorporated via adsorption from an alkaline solution; the 5,10,15,20-tetrakis(4-aminophenyl)porphyrin (TPP-TetraNH_2_), due to its insolubility in aqueous alkali, was incorporated into chitosan via dissolution and homogenization in mixed solvents (aqueous acetic acid, chloroform) followed by casting. Meanwhile, the zinc(II) phthalocyanine tetrasulfonic acid (ZnPcS) was covalently attached to the amino functions after being converted into the corresponding sulphonyl chloride. The absorption spectrum of the different materials was an important tool to confirm and to estimate the amount of the different dyes incorporated into the polymeric membrane. A preliminary evaluation of their photomicrobicidal activity in static systems against *E. coli* allowed for the selection of the phthalocyanine/chitosan membrane as the most effective one, showing no detectable microorganisms after 30 min under irradiation with a halogen lamp (500 W, 230 V, 50 Hz). In the photomicrobicidal studies using the circulating water photoreactor system designed by the authors, the nylon reinforced ZnPcS/chitosan membrane showed a significant photoinactivation (>2 log) of *E. coli* present at 10^5^ cells mL^−1^. The authors highlighted that the photodynamic effect was maintained (although at a reduced level) after the membrane had been kept in the dark for 9 months.

**Figure 18:**
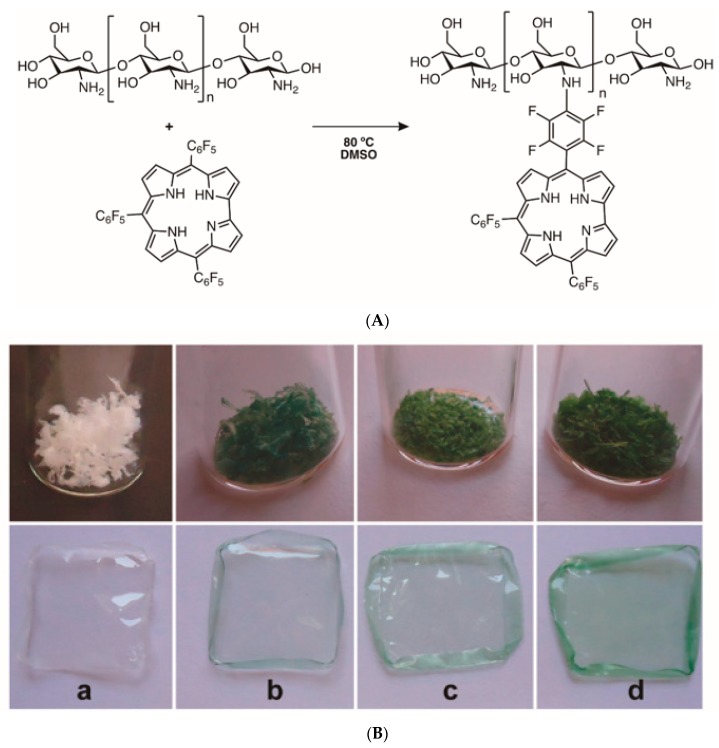
(**A**) Conditions used to graft 5,10,15-tris(pentafluorophenyl)corrole in chitosan. (**B**) Visual appearance of chitosan (**a**) and of corrole grafted-chitosan derivatives obtained after different reaction times [(**b**) 6 h, (**c**) 24 h, and (**d**) 48 h], and of the corresponding films. Reproduced from Reference [30] with permission from the American Chemical Society.

Based on the easy replacement of the fluorine atom in the *p*-position of pentafluorophenyl units by nucleophiles [112,113], Barata et al. [30] reported the synthetic access to transparent and fluorescent chitosan films bearing corrole units obtained from 5,10,15-tris(pentafluorophenyl)corrole with bacteriostatic action. The graft of 5,10,15-tris(pentafluorophenyl)corrole to chitosan was performed in dimethyl sulfoxide at 80 °C (Figure 18A,B) and it was verified that the amount of corrole linked to chitosan was dependent on the reaction time. Additionally, it was proved that the presence of the corrole units did not affect the film-forming ability and all films with good thermomechanical properties and thermal stability showed a bacteriostatic effect even in the dark against *S. aureus* (reduction of 2 log).

**Figure 19:**
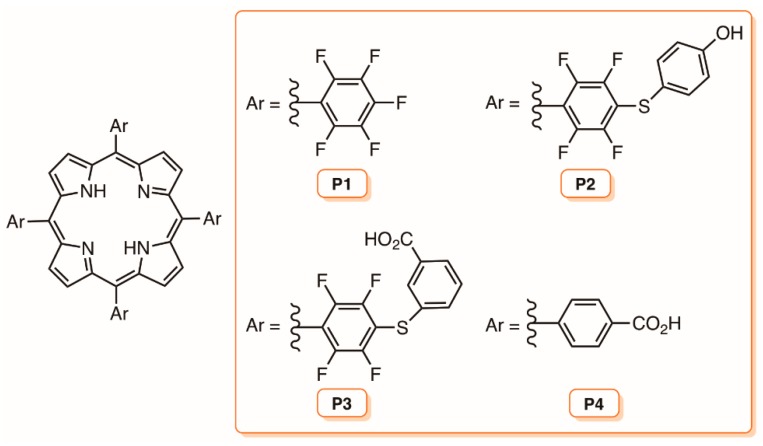
Structure of *meso*-tetraarylporphyrin P1–P4, bearing phenyl or pentafluorophenyl groups at the *meso* positions with or without acid groups used to prepare chitosan films (adapted from Reference [109]).

Considering the suitable features of chitosan to act as a support for PS as well as its antimicrobial activity and film forming ability, Neves and colleagues reported the synthetic access to new materials obtained through the non-covalent incorporation of *meso*-tetraarylporphyrins P1–P4 bearing phenyl or pentafluorophenyl groups at the *meso* positions with or without acid groups in chitosan (Figure 19) [109]. The effectiveness of the non-immobilized porphyrins and of the porphyrinic-chitosan films (PS-CF) to photoinactivate *Listeria innocua* and to prevent *L. innocua* biofilm development was evaluated and it was verified that the photodynamic inactivation was dependent on the porphyrin structure and also on their ability to produce ^1^O_2_. The biofilm development was almost absolutely inhibited in the presence of PS-CFs containing porphyrins P1 and P2 after being irradiated with white light for 24 h (irradiance of 10 mW cm^−2^) followed by incubation in the dark for 48 h. The authors highlighted that the good results associated with the easy recovery of the films merit further investigation using other PSs and new biofilm-forming microorganisms with potential application as an anti-fouling coating materials for the food industry [109].

Considering the potential of the PDI approach to treat infected dental hard tissues in a clinical scenario [114,115,116], but also under other contexts, several groups evaluated the antibacterial performance of conjugates involving dyes like Rose Bengal and methylene blue and chitosan [117,118,119,120,121]. For instance, Kishen and co-workers [120] reported the photodynamic efficacy against dental biofilms of the conjugate Rose Bengal-chitosan (CS-RB) obtained from the reaction between RB and chitosan in the presence of the coupling agent EDC (*N*-ethyl-*N*′-(3-dimethylaminopropyl)carbodiimide). The photodynamic efficacy of CS-RB, with a high efficiency to generate ^1^O_2_, was evaluated towards planktonic and in vitro biofilms of *E. faecalis* after activation with green light (540 nm) using light doses between 5 to 60 J cm^−2^. The results indicated that the in vitro CS-RB against *E. faecalis* in planktonic cells gave a much higher inactivation, either in the dark or in the presence of light, when compared with RB alone (>7 log of reduction) in both light regimes. However, in the presence of *E. faecalis* biofilms, the bacterial reduction was not so drastic, achieving in both cases (CS-RB and RB alone) a reduction of at least 3 log after photodynamic treatment. Nevertheless, when CS-RB was employed, the biofilm photoinactivation was light-dose dependent and was also significantly higher than that by RB at 40 and 60 J cm^−2^. Moreover, in the assays concerning cytotoxicity and photocytotoxicity in fibroblasts cells, it was found that the presence of CS-RB gave higher fibroblast cell survival than did RB alone. Additionally, the dentin collagen tests used to evaluate the chemical changes, resistance to enzymatic degradation, and mechanical properties demonstrated that the CS-RB-cross-linked dentin collagen showed higher resistance to collagenase degradation and superior mechanical properties. Thus, it was envisaged that the photoactivated CS-RB conjugate could be a targeted treatment strategy to deal with infected dental hard tissues in a clinical scenario, where both disinfection and structural integrity need to be concomitantly addressed.

**Figure 20:**
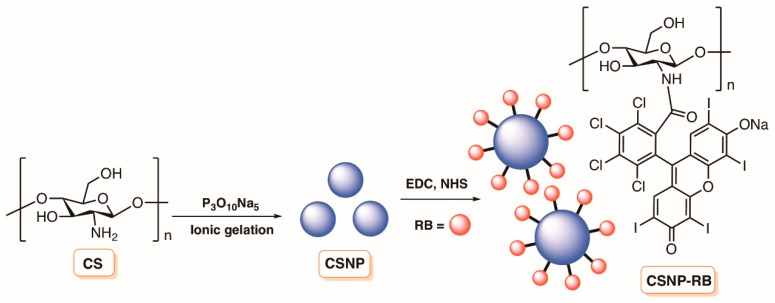
Conditions used to graft Rose Bengal in CSNP (adapted from Reference [121]).

As an extension of the previous work, the same group [121] developed NPs based on polymeric chitosan covalently linked to Rose Bengal (CSNP-RB) (Figure 20) and evaluated their efficacy in reducing the viability of *E. faecalis* biofilms to disrupt biofilm structure and their ability to stabilize the dentin-collagen structural integrity. The chitosan nanoparticles (CSNP) were prepared by treating chitosan, previously dissolved in acetic acid, with a solution of sodium hydroxide in order to increase the pH to ≈5 and then with sodium tripolyphosphate under stirring. The NPs were obtained by centrifugation at 20,000 rpm for 30 min followed by the conventional work-up. The conjugation of CSNP to RB acid group was performed in the presence of EDC and *N*-hydroxysuccinimide (NHS), as previously described. The study showed that the CSNP-RB was also less toxic to fibroblasts and had significant antibacterial activity (reduction > 8 log) even in the presence of bovine serum albumin. The authors explained that CSNP-RB exerted an antibacterial mechanism by adhering to a bacterial cell surface, permeabilizing the membrane and lysing the cells subsequent to photodynamic treatment. Incorporation of CSNP-RB and photocrosslinking significantly improved resistance to degradation and the mechanical strength of dentin-collagen.

Interestingly, in 2016, Mashayekhan and co-workers reported that the presence of chitosan nanoparticles (CSNPs) could improve the photodynamic action of methylene blue towards *S. aureus* and *P. aeruginosa* biofilms, and human fibroblasts [122]. The CSNPs were prepared using the previous ionic gelation method and the dynamic light scattering (DLS), and field-emission scanning electron microscope (FESEM) results confirmed their nanometric size. The photodynamic assays performed in the presence of MB + CSNPs at a final concentration of 50 µM MB and an irradiance of 22.93 J cm^−2^ showed a significant anti-biofilm photoinactivation (>3 and >2 log CFU reduction in *S. aureus* and *P. aeruginosa* biofilms, respectively) while MB alone led to a reduction of approximately < 1 log CFU. At the same experimental conditions, only 25.1% of the fibroblasts were photoinactivated by MB + CSNPs and the efficacy of MB-PDI was explained by considering that the polycationic CSNPs were able to disrupt biofilm structure allowing a deeper and higher penetration of MB into the biofilms.

**Figure 21:**
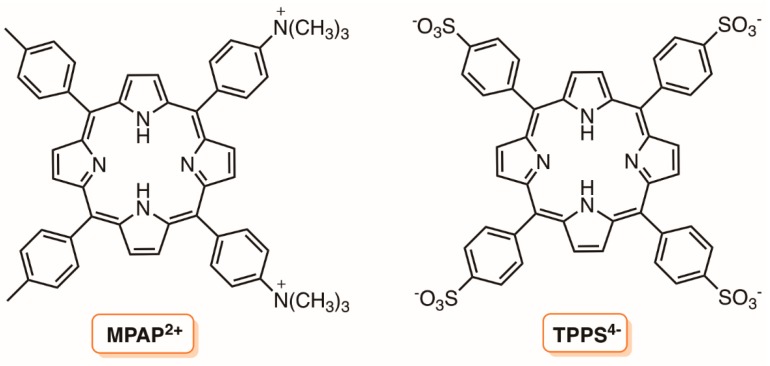
Structures of 5,10-di(4-methylphenyl)-15,20-di(4-*N,N,N*-tri-methylammoniumphenyl)porphyrin (MPAP^2+^) and 5,10,15,20-tetrakis(4-sulphonatophenyl)porphyrin (TPPS^4−^) (adapted from Reference [123]).

In a previous study, Durantini and co-workers [123] also reported the effect of chitosan (CS) and other additives (divalent cations and EDTA) on the uptake and on the photoinactivation of *E. coli* in the presence 5,10,15,20-tetrakis(4-*N*,*N*,*N*-trimethylammoniumphenyl)porphyrin (TAAP^4+^) (Figure 2), 5,10-di(4-methylphenyl)-15,20-di(4-*N,N,N*-tri-methylammoniumphenyl)porphyrin (MPAP^2+^), and 5,10,15,20-tetrakis(4-sulphonatophenyl)porphyrin (TPPS^4−^) (Figure 21). The authors verified that the addition of Ca^2+^ or Mg^2+^ to the cultures enhanced the cell uptake of MPAP^2+^ and TPPS^4−^, but not of TMAP^4+^. Concerning the other two additives, EDTA produced an increase in the uptake of all porphyrins, while CS mainly enhanced the amount of TPPS^4−^ bound to *E. coli*. In the presence of Ca^2+^ and Mg^2+^, the phototoxic activity mediated by MPAP^2+^ and TPPS^4−^ towards *E. coli* was increased but was reduced in the presence of TMAP^4+^. EDTA did not affect the photoinactivation induced by cationic porphyrins, while a small enhancement was found for TPPS^4−^. In the presence of CS, using a slightly toxic CS concentration, an enhanced photoinactivation of *E. coli* by TPPS^4−^ was observed, contrary to what was observed with TMAP^4+^. Therefore, the combined cytotoxic activities of CS and PDI mediated by TPPS^4−^ could be beneficial for the inactivation of *E. coli*.

### 4.2. Cellulose

Cellulose is the most abundant natural biopolymer, consisting of high molecular weight β-1,4-linked anhydro-d-glucose polymer chains, and therefore, is also considered a great starting material for developing new and more sustainable materials from renewable resources. As cellulose owns a carbohydrate nature, it has inherent compatibility with biological tissues and, consequently, possesses unique utility with respect to their bioavailability, biocompatibility, and biodegradability considerations.

Considering that cellulose could be an excellent material to develop germicidal surfaces at low cost, in 1994, Bonnett and Galia [124] reported the photobactericidal properties of “cellophane” films (regenerated cellulose) impregnated with the cationic porphyrins 5,10,15,20-tetrakis(1-methylpyridinium-4-yl)porphyrin tetra-tosylate (TMPyP) and with 5,10,15,20-tetrakis(*N*,*N*,*N*-trimethylanilinium-4-yl)porphyrin tetra-tosylate (TAAP^4+^) (Figure 2). The impregnation time required was performed by maintaining commercially available cellophane sheets of 50 µm thickness in contact with the porphyrins dissolved in aqueous solution or aqueous methanol solution at about 50 °C. The amount of absorbed porphyrin was estimated by UV-Vis spectroscopy after washing thoroughly. The authors verified that the films containing TMPyP, after exposure to a 1500 W xenon lamp for 50 h, retained their flexibility and strength and were not photobleached (although some photochemical changes occurred such as a slightly opacity). No bacterial growth was verified when the films impregnated with TMPyP (18 µg cm^−2^) were maintained in contact with bacterial inoculum of *S. aureus, Proteus vulgaris* and *E. coli* for 24 h under visible light (fluorescent lamp) irradiation. Similar photobactericidal results were reported with the TAAP^4+^ only towards *S. aureus.* The results with *P. aeruginosa* strain were not conclusive. The authors highlighted that the attempts to impregnate the cellophane films with anionic porphyrins were not successful.

In 2016, Rassa and co-workers [125] also selected the tetracationic porphyrin TAPP^4+^ (Figure 2) and the corresponding Zn(II) complex to prepare cellulosic fabrics for antimicrobial applications. The impregnation was performed by soaking a 100% cellulosic fabric, pretreated with an aqueous solution of Na_2_CO_3_ at 50 °C for 30 min, with solutions of the porphyrins in PBS. After 30 min at 50 °C, the unbound porphyrin was thoroughly washed, and the molar grafting ratio was calculated using UV-Vis. The basic pretreatment was important to activate the cellulose hydroxyl group in order to promote electrostatic interactions with the cationic PS. The authors noted that the grafting yield was dependent on the initial concentration of the PS and its presence in the cellulosic fabrics was confirmed by spectroscopic techniques. The antimicrobial activity of the new materials was tested under visible light irradiation (100 W tungsten lamp with an average intensity of ≈0.36 mW cm^−2^) towards *S. aureus*, *P. aeruginosa*, and *E. coli.* The results showed that the irradiation period, the PS concentration, and the bacterial strain were important factors to be taken in account. *S. aureus* was fully inactivated with both prepared cellulose fabrics at a PS concentration of 100 µM after 30 min of irradiation, while for *P. aeruginosa*, at the same PS concentration, the full inactivation was only attained in the presence of the ZnTAPP^4+^ after 90 min of irradiation. The worse efficiency of the materials towards *E. coli* (58.5% for TAPP^4+^ and 30% for ZnTAPP^4+^) at the same concentration and after 90 min of irradiation was explained by considering the more complex composition of this Gram-(-) cell wall, that seems to protect this strain more effectively from extracellular ^1^O_2_.

**Figure 22:**
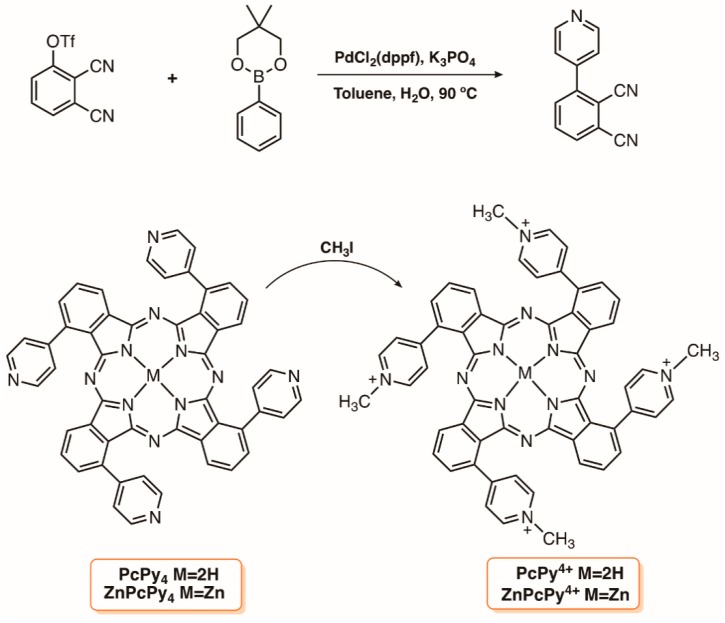
Synthetic access to the phthalonitrile used in the preparation of phthalocyanines (Zn)PcPy_4_ and (Zn)PcPy^4+^(adapted from Reference [126]).

The cationic phthalocyanines PcPy^4+^ and ZnPcPy^4+^ with four pyridyl substituents linked via a direct C-C bond to the α-phthalo-positions (Figure 22) were also considered in the design of photoactive antimicrobial surface based on cellulose [126]. The synthetic access to those derivatives required the previous preparation of the pyridine phthalonitrile from the pyridine boronate ester and the triflate phthalonitrile (Figure 22). Then, tetramerization under conventional conditions to afford the neutral precursors PcPy_4_ and ZnPcPy_4_ followed by methylation with iodomethane in DMF gave rise to the desired cationic salts (PcPy^4+^ and ZnPcPy^4+^). The antimicrobial surfaces were prepared by soaking 3.5 × 3.5 cm filter papers (Whatman 1) in aqueous solutions containing the neutral and the cationic dyes at different concentrations (range from 0.008 to 0.08 mg cm^−2^). The photodynamic antimicrobial efficacy of the dyed filter paper samples using bioluminescent *E coli* and *Acinetobacter baylyi ADP1* strains allowed for the selection of the zinc complex ZnPcPy^4+^ as the best PS; the PDI experiments were performed for 60 min using white light (wavelength 485–750 nm) of intensity 18 mW cm^−2^. The antimicrobial activity of the filter paper dyed with ZnPcPy^4+^ achieved a reduction of 2.7 log CFU against *E. coli* and of 3.4 log against *A. baylyi.*

The preparation of photoactive materials based on the non-covalent interaction of phenothiazine dyes like TBO and RB (Figure 2) with cellulose also deserved some attention from the scientific community. For instance, Michael Wilson [127] evaluated the efficacy of cellulose acetate containing the TBO towards MRSA and *P. aeruginosa* under light conditions similar to those present in hospitals (white light, 60 W domestic lamp bulb, for up to 24 h). The antimicrobial coatings were prepared just by adding the TBO to a solution of cellulose acetate in acetone and then leaving the solvent to evaporate. The approach was considered to have the potential to minimize the transmission of infectious diseases through surfaces in hospitals since substantial bacterial reduction (10^5^ CFU cm^−2^) was achieved following irradiation of the film.

In 2006, the same group [128] evaluated the efficacy of the previous material and also of an analogue containing RB towards microorganisms such as *S. aureus* and MRSA, *E. coli*, *C. albicans, Clostridium difficile*, and bacteriophage *ØX174*. The results confirmed the high efficacy of the doped films upon illumination with a white light emitted from a 28 W fluorescent lamp for periods between 2 to 16 h, with bacterial reductions ranging from 6.3 to 6.7 log. *C. albicans* was found to be the least sensitive microorganism to the material photodynamic action (0.9 log reduction after 16 h irradiation).

Considering that aerosols are important vehicles for the transmission of infectious diseases in hospital setting, the same group still evaluated the efficacy of TBO and RB on cellulose acetate towards *S. aureus* from aerosols [129]. The assays were performed by spraying the photoactive surfaces with *S. aureus* (10^5^ CFU m^−2^) suspended in phosphate-buffered saline, saliva, or horse serum. The inactivation ranged from 0.8 log (in horse serum) to 2.5 log (in PBS) after 6 h of exposure with white light emitted from a 28 W domestic fluorescent lamp.

**Figure 23:**
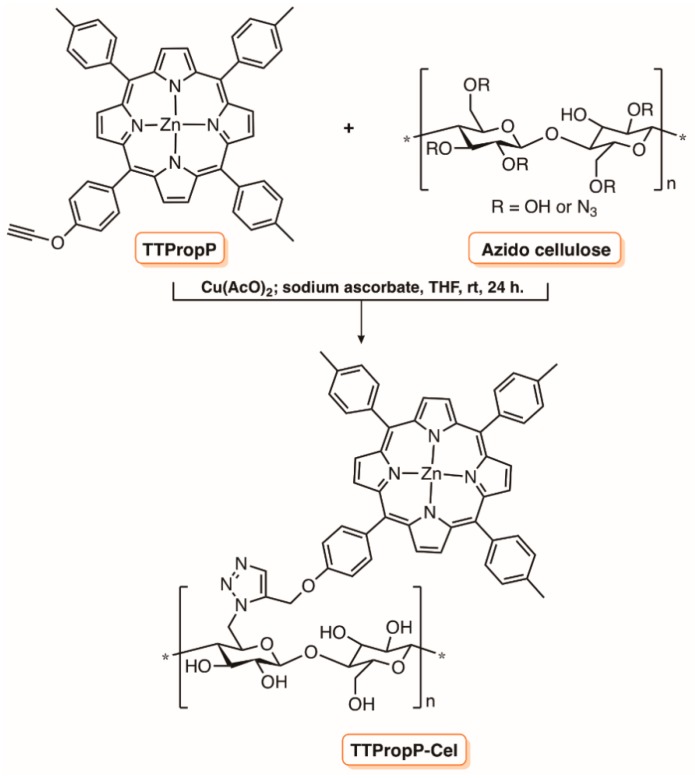
Synthetic methodology used to covalently graft the acetylenic porphyrin TTPropP in azido cellulose fabric (adapted from Reference [130]).

The possibility to prepare photoactive cotton fabrics by covalently linking the PS to cellulose was also considered in different studies [130]. For instance, Krausz and co-workers [130] reported the synthetic access to a new antibacterial material, TTPropP-Cel, by grafting through a “Click-Chemistry” approach the acetylenic porphyrin derivative TTPropP on cellulose functionalized with azide units (Figure 23). The authors found that under irradiation with visible light, the material displayed antibacterial activity against representative strains of *E. coli* and *S. aureus*.

**Figure 24:**
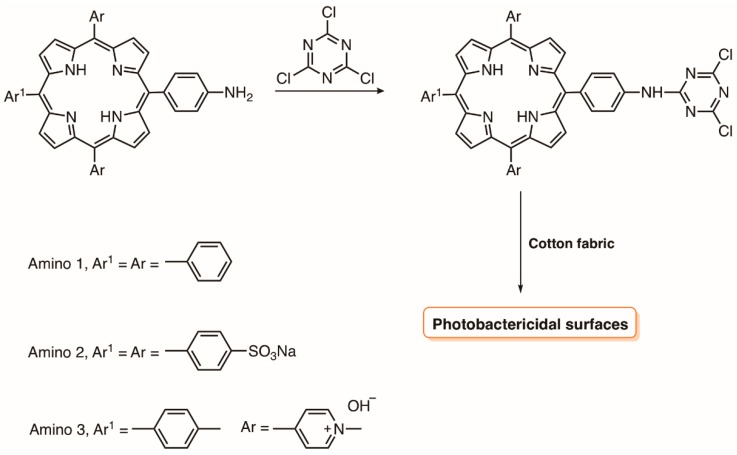
Synthetic methodology used to covalently graft the amino porphyrins 2-4 on cotton fabrics via 1,3,5-triazine linker (adapted from Reference [131]).

Soon after, the same group [131] reported the covalent grafting of the neutral, anionic and cationic amino 1–3 porphyrins on cotton fabrics via a 1,3,5-triazine linker without any preliminary chemical modification of the cellulosic material (Figure 24). When subjected to light irradiation of 0.16 mW cm^−2^ for 24 h (total light dose 13.8 J cm^−2^), the cationic fabric exhibited the best performance (100% photoinactivation against *S. aureus* while no photoactivity was detected against *E. coli*).

**Figure 25:**
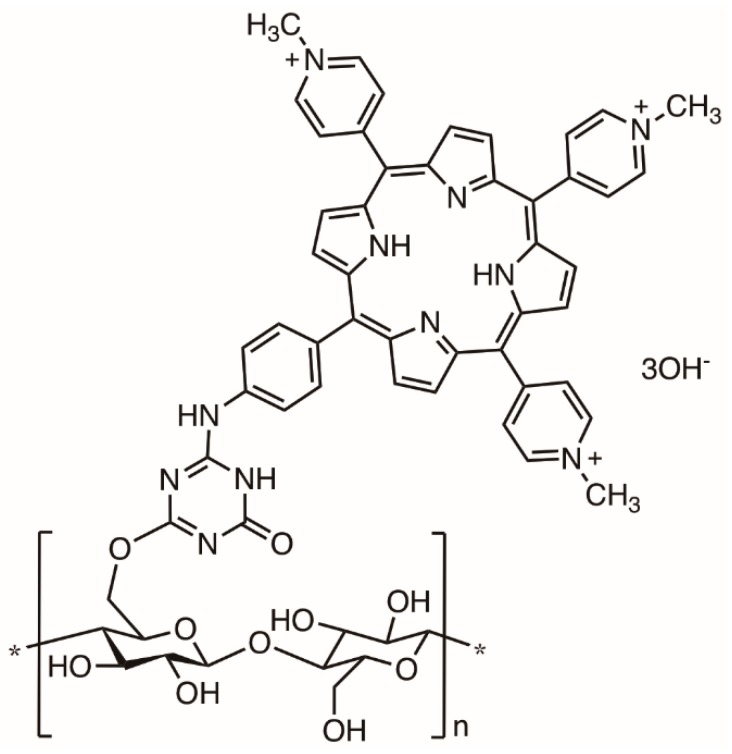
Structure of the antimicrobial filter paper bearing a tricationic porphyrin (adapted from Reference [132]).

A better efficiency towards *E. coli* was found when the authors tested the photobactericidal surface, obtained by grafting a tricationic porphyrin in filter paper through the 1,3,5-triazine linker (Figure 25). The authors verified that the untreated paper in the dark or under irradiation and the treated paper in the dark allowed a bacterial growth of 4 and 2 log units for *S. aureus* and *E. coli*, respectively, when compared with the initial bacterial concentration. However, no survival of either type of bacteria was detected on the paper bearing the porphyrin after light exposure for 24 h, totaling a light dose of 9.5 J cm^−2^ [132].

**Figure 26:**
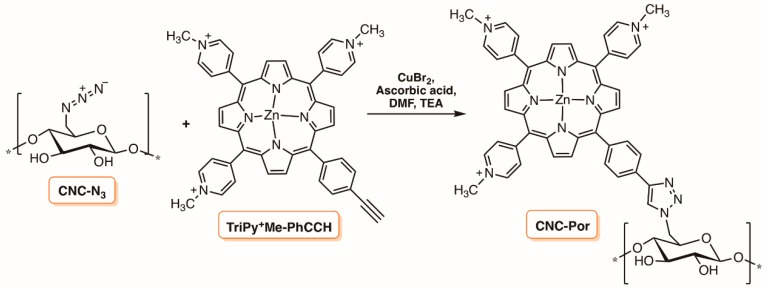
Preparation of a cellulose nanocrystal conjugated to a cationic porphyrin TriPy^+^Me-PhCCH (adapted from Reference [133,134]).

The Cu(I)-catalyzed Huisgen 1,3-dipolar cycloaddition was also selected by Ghiladi and co-workers to append the tricationic porphyrin TriPy^+^Me-PhCCH bearing an alkynyl function to cellulose nanocrystals containing azide moieties (Figure 26) [133,134]. It was commented that cellulose nanocrystals (CNC) possessed some benefits against amorphous cellulose ester due to their defined surface structure and dimension and a particular degree of molecular control for precise functionalization because of their rigidity. The authors prepared the cellulose nanocrystals by hydrolysis of Whatman #1 filter paper (98% α-cellulose, 80% crystallinity), previously blended using aqueous HBr. After removing the excess of acid, water-soluble fragments and ultrafine particles of CNC were obtained with an average length of 100–400 nm. The nanocrystals were then functionalized with the azide moities for a further reaction with the porphyrin TriPy^+^Me-PhCCH. Although, only suspended in an aqueous system, the CNC-Por showed excellent efficacy toward the photodynamic inactivation of *Mycobacterium smegmatis* and *S. aureus*, albeit with only a slight activity against *E. coli*.

In the first study, the bacterial cultures (≈10^8^ CFU mL^−1^) were irradiated with white light (400–700 nm; 60 mW cm^−2^) in PBS with 20 µM of CNC-Por after a dark incubation period and the results showed a 6 log reduction in viable cells against *S. aureus* (5 min incubation, 30 min irradiation), 3.5 log for *M. smegmatis* (45 min incubation, 30 min irradiation), and 2 log for *E. coli* (60 min incubation, 30 min irradiation) [133]. In the second study, the efficacy of the photoactive CNC-Por conjugate, also at 20 µM, was tested against *A. baumannii*, multidrug resistant *A. baumannii* strain, MRSA, and *P. aeruginosa* (≈10^8^ CFU mL^−1^) with visible light (65 mW cm^−2^) [134]. A decrease of 6 log units in viable cells was observed for MRSA (5 min incubation), 5–6 log units for *A. baumannii* (30 min incubation) and multidrug resistant *A. baumannii* (15 min incubation), and 2.5 log units for *P. aeruginosa* (60 min incubation) at a total light dose of 118 J cm^−2^ (30 min irradiation). Confocal laser scanning microscopy analysis of CNC-Por after incubation with *A. baumannii* or *S. aureus* suggested a lack of internalization of the PS. Considering both studies, the authors commented that CNC-Por was able to mediate the photodynamic inactivation of all the tested bacterial strains studied. Confocal microscopy demonstrated that the mode of action for CNC-Por did not proceed through a PS-binding or uptake mechanism. Rather, the cytotoxic species (e.g., ^1^O_2_ and other ROS) generated by the photodynamic process were likely to diffuse over short distances to ultimately damage the bacteria, leading to cell inactivation as already stated in previous studies [135]. The authors commented the potentiality of the prepared materials to reduce the rates of transmission of a wide range of bacteria, particularly antibiotic resistant strains, which is important for hospitals and healthcare-related industries [134].

**Figure 27:**
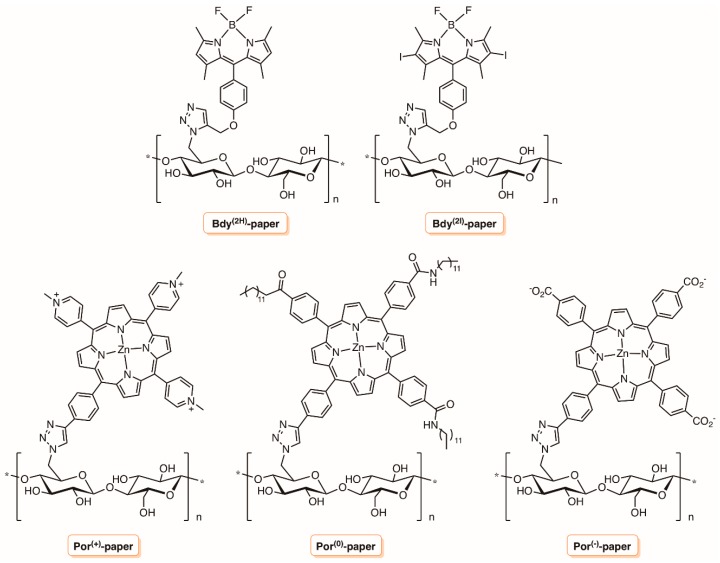
Structure of the antimicrobial filter paper bearing different porphyrins and BODIPY (adapted from Reference [136]).

Carpenter et al. [136] also employed cellulose paper activated with azide moieties to graft differently-charged porphyrins and boron dipyrromethenes (BODIPY) bearing an alkenyl unit via the “Click-Chemistry” approach (Figure 27). The bactericidal papers obtained with a dye load of ≈8 mg g^−1^ (12.4 nmol mg^−1^, MW ¼ 672) were fully characterized and their antibacterial efficacy was evaluated against *S. aureus*, vancomycin-resistant *E. faecium*, *A. baumannii*, *P. aeruginosa*, and *Klebsiella pneumoniae*. The best results were obtained with the Por^(+)^-paper conjugate that was able to photoinactivate >4 log (99.99+%) of all bacterial strains studied upon illumination for 30 min with white light (65 mW cm^−2^, 400−700 nm). The same Por^(+)^-paper was also able to photoinactivate dengue-1 virus by >4 log, influenza A virus by ≈2.5 log (~99.5%) and human adenovirus-5 by ≈2 log (≈99%).

**Figure 28:**
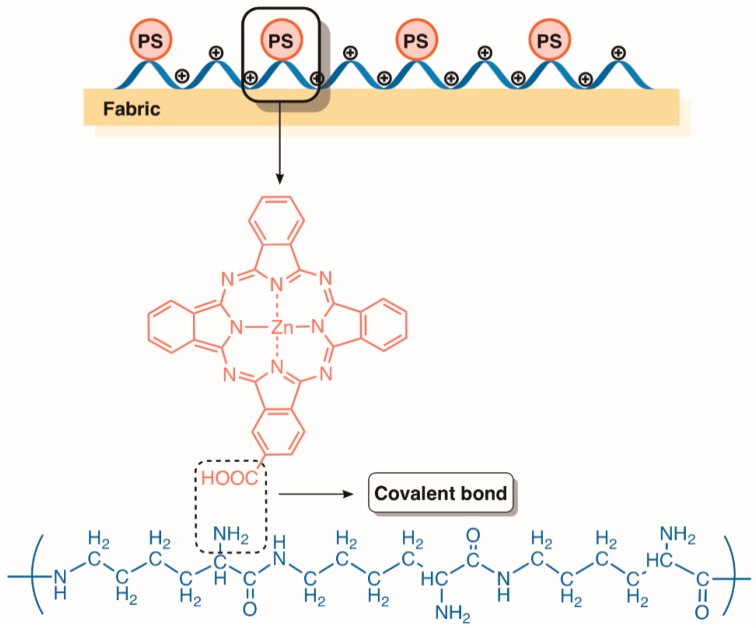
Design strategy of PS-ε-polylysine-fabric material with dual antimicrobial mechanisms (adapted from Reference [137]).

Considering the requirement of antimicrobial textile products, such as gowns, beddings and wound dressings for the health industry, in order to decrease the occurrence of hospital-acquired infections [137], several synthetic and natural fibers have been manufactured in the presence of antimicrobial products. For instance, cellulose has been modified into antimicrobial wound dressings by introducing numerous antimicrobial or disinfecting agents such as antibiotics, peptides, polymers, and inorganic nanoparticles [138]. Under this context, Chen and colleagues concentrated their efforts in developing fabrics with antimicrobial features. The group found out that the efficacy of a fabric only covered with the antimicrobial agent ε-polylysine could be improved by adding a second layer of a PS agent (the zinc(II) complex of a mono-substituted β-carboxyphthalocyanine) onto the fabric (Figure 28). Therefore, the positively charged bottom layer of ε-polylysine was able to disrupt the bacterial membrane, while the zinc phthalocyanine derivative was able to inactivate microorganisms by ^1^O_2_ and other ROS, thus compensating the ε-polylysine low antimicrobial efficacy. The authors found that the fabric was stable, durable, and washable if ε-polylysine was first adsorbed before the condensation between the present PS acid groups and the amino groups onto ε-polylysine under mild conditions. This special fabric proved to be a potent antimicrobial against both Gram-(−) and Gram-(+) bacterial strains, decreasing the survival of *E. coli* or *S. aureus* by 99% and 98%, respectively. Besides, this fabric was also able to kill a drug resistant bacterial strain. At the molecular level, the authors found that conjugated PS exists in both monomeric and aggregate forms, the former accounting for its antimicrobial efficacy, while the latter was responsible for the photostability of the fabric. Consequently, this work demonstrated the feasibility of double coatings with dual antimicrobial mechanisms on the material surface to kill bacteria, which can be used in other antimicrobial materials [137].

**Figure 29:**
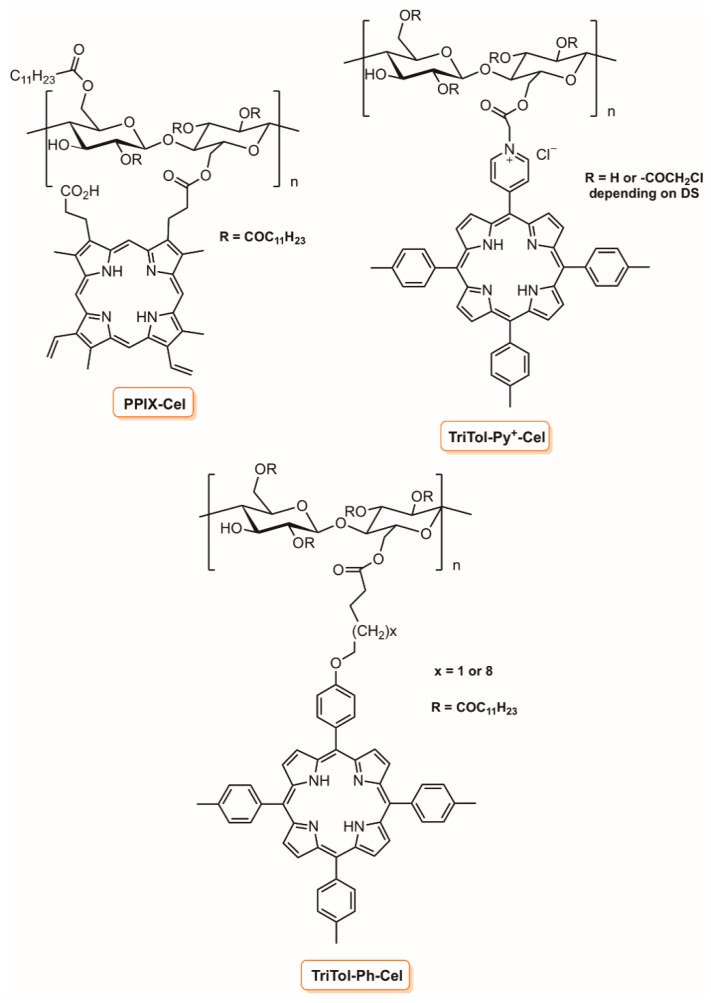
Structures of photobactericidal plastic films based on esterified cellulose with fatty acids and porphyrin (adapted from Reference [139,140]).

Krausz and co-workers [139,140] developed photobactericidal plastic films based on esterified cellulose with fatty acids and porphyrins (Figure 29). In their first report, they considered plastic films obtained by esterification of cellulose with protoporphyrin IX and lauric acid (PPIX-Cel), while in the posterior works, they selected adequately substituted *meso*-tetraarylporphyrins (TriTol-Py^+^-Cel, TriTol-Ph-Cel) with acid groups, namely a cationic one (Figure 29). The biological results towards *E. coli* and *S. aureus* strains showed that the photobactericidal activity of the plastic films prepared was dependent on the porphyrin grafting percentage, and in the absence of porphyrin, the films allowed the full growth of bacteria.

### 4.3. Superparamagnetic Iron Oxide Nanoparticles (SPION) Functionalized with Dextran

Superparamagnetic iron oxide nanoparticles (SPIONs) are a class of NPs that offer a variety of applications, comprising magnetic resonance imaging, drug delivery, cell labeling, tissue targeting, tissue repair, and hyperthermia [141,142,143]. They are found in different forms in nature such as hematite (α-Fe_2_O_3_), maghemite (γ-Fe_2_O_3_), magnetite (Fe_3_O_4_), among other forms. However, the most studied SPIONs are γ-Fe_2_O_3_ and Fe_3_O_4_ [142,144].

As SPIONs have a certain propensity to agglomerate, their surfaces have to be adjusted with biocompatible molecules and/or polymers in order to obtain stable NPs in physiological media [144]. They can be functionalized with hydrophilic ligands such as polyethylene glycol (PEG), dextran, phospholipids, chitosan, and catechol derivatives [145]. Therefore, these ligands can be used in combination with PSs to produce a water-stable SPION that can be used in the photodynamic approach. In fact, several groups prepared SPIONs that were stable in water containing PSs, such as chlorin e_6_ [146], hematoporphyrin monomethyl ether [147], or a dendrimer porphyrin derivative [148]. Additionally, as SPIONs are easily manipulated by an external magnetic field, they can then be directed to the location of infection and where the antimicrobial agent can be activated, leading to the inactivation of microbial cells, with the tiniest side effects for the host [144]. Besides, due to their superparamagnetic features, SPIONs can also be employed to isolate contaminants from wastewater [149].

**Figure 30:**
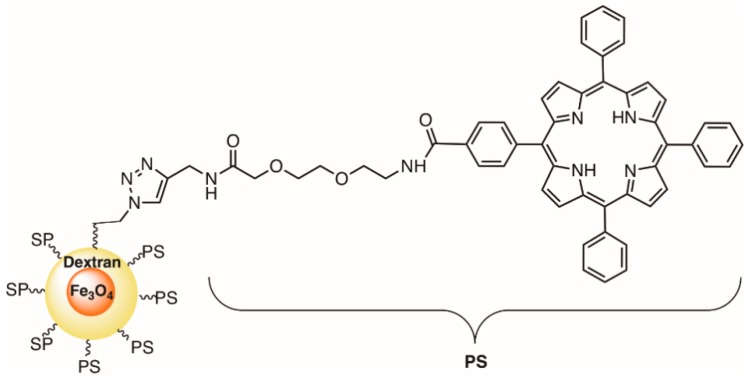
Structure of the SPION-MCTPP (adapted from References [150,151]).

Considering the promising results obtained with the use of TCPP incorporated in SPIONs as PS towards murine melanoma cells [150], Thandu et al. decided to evaluate their potential in the photoinactivation of bacteria [151]. Therefore, in 2017, Thandu et al. [151] reported the use of SPION-MCTPP obtained from 5-(4-carboxyphenyl)-10,15,20-triphenylporphyrin (MCTPP) (Figure 30) against *S. aureus, Streptococcus mutans*, and *E. coli*. It was observed that when exposed to blue light (470 nm) at an irradiance of 4.8 mW cm^−2^, SPION-MCTPP at 0.5 µM caused an inactivation of *S. aureus* of 2 log after 180 min of irradiation. For *S. mutans*, only a 1 log decrease was observed under the same experimental conditions. This nanoconjugate was not effective against the Gram-(−) bacterium *E. coli*. The possibility of recovering this nanoconjugate with magnets, post treatment, was an important advantage when it is considered for disinfection applications [151].

## 5. Liposomes

**Figure 31:**
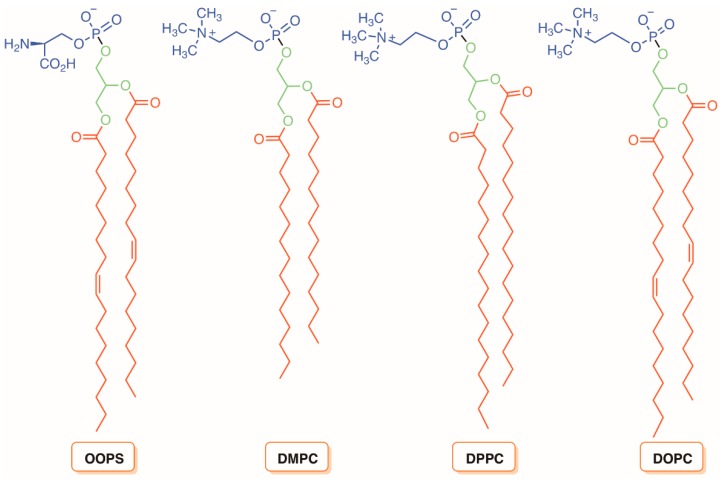
Structure of phospholipids l-α-dioleoylphosphatidylserine (OOPS), l-α-dimiristoylphosphatidylcholine (DMPC), l-α-dipalmitoylphosphatidylcholine (DPPC), and 1,2-dioleoyl-*sn*-glycerophosphatidylcholine (DOPC) (adapted from Reference [69]).

The hydrophobic nature of most PSs makes them aggregate in aqueous medium, thus diminishing the PDI efficacy because it reduces the ^1^O_2_ formation by a self-quenching effect in the excited state [152]. Thus, in order to preserve the monomeric state of the PS, to protect it from aqueous environment, and to increase the efficacy of photodynamic treatment, several transporters and delivery systems, such as liposomes, have also been developed in the last few years. Liposomes became known due to their high loading capacity, biodegradability, biocompatibility, and size [153]. In fact, these liposomal vesicles with one or more concentric phospholipid bilayer(s) have two main advantages in PDI (Figure 31).

First, they can accommodate both hydrophobic and hydrophilic PSs and thus can be used to inactivate microorganism cells after irradiation with light. Second, the synergistic effect of positively charged and highly fluid components of the liposomes increases the PS uptake by microorganisms, as well as the overall phototoxicity [154,155,156]. The release of liposome content can be accomplished in several ways involving photochemical activation (photoisomerization, photocleavage, and photopolymerization), as well as by photophysical activation, such as photothermal conversion [157]. In addition, the release rate of the drug incorporated into liposomes depends on the drug itself and the properties of the liposome such as composition, temperature, sensitivity to changes with the pH, and osmotic gradient. Microwave radiation and pulses of laser light can also lead to the release of drugs from liposomes [157,158]. The possibility of the release of other drugs incorporated into liposomes using a laser light can be exploited in combination with PDI.

**Figure 32:**
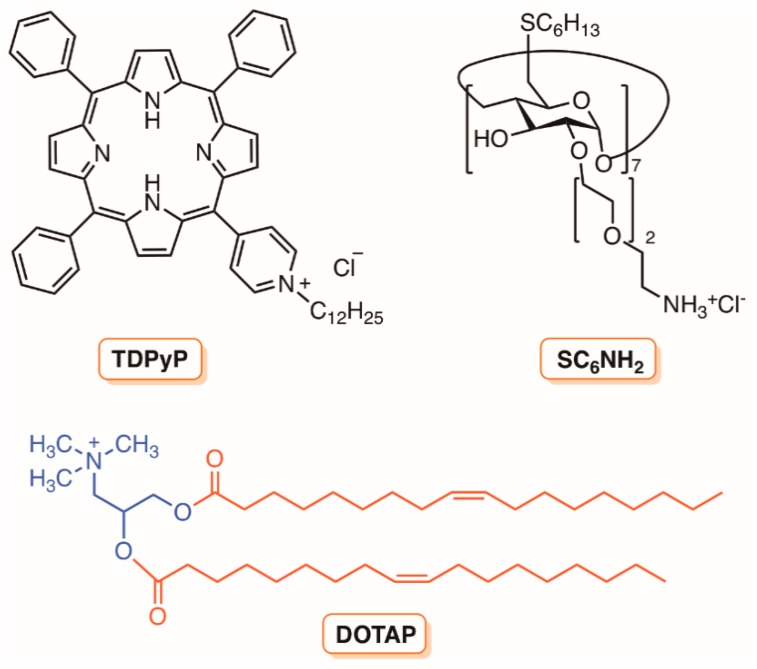
Structure of 5-(1-dodecanoylpyridinium-4yl]-10,15,20-triphenylporphyrin (TDPyP) and the cationic deliver systems DOTAP and SC_6_NH_2_ (adapted from Reference [160]).

In 2007, Jori and co-workers [159] compared the photodynamic action of the monocationic porphyrin 5-(1-dodecanoylpyridinium-4yl]-10,15,20-triphenylporphyrin (TDPyP) (Figure 32) against a bacterial MRSA strain in a homogeneous aqueous solution, and after being incorporated into neutral phospholipid vesicles of dl-α-dipalmitoylphosphatidylcholine (DPPC) and l-α-dimiristoylphosphatidylcholine (DMPC), or into the cationic liposome of *N*-[1-(2,3-dioleoyloxy)propyl]-*N*,*N*,*N*-trimethylammonium chloride (DOTAP).

The results showed that the low photosensitizing activity (<1 log after 10 min of white light irradiation) of this PS (with high ability to generate ^1^O_2_) in aqueous solution or in neutral lipid vesicles was dramatically improved (>6 log) after its incorporation in the polycationic liposome. This behavior change of photinactivation was attributed to the action of cationic liposome (DOTAP) disorganizing the bacterial wall, thus enhancing its permeability to the PS molecules. A similar effect towards MRSA and *E. coli* was found when the same porphyrin derivative was incorporated into the cationic amphiphilic heptakis [2-(ω-amino-*O*-oligoethyleneglycol)-6-deoxy-6-hexylthio]-β-cyclodextrin (SC_6_NH_2_) (Figure 32) [160].

**Figure 33:**
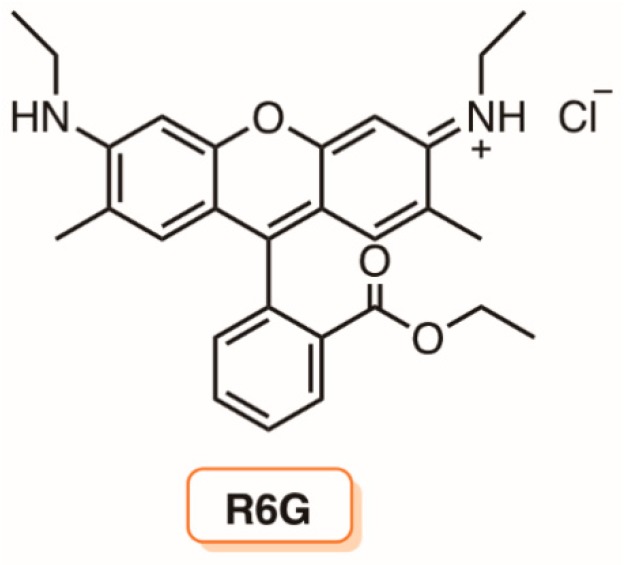
Structure of Rhodamine 6G that was encapsulated in liposomes (adapted from Reference [161].

The improvement of the photodynamic inactivation using a PS stabilized in positively charged liposomal formulations was corroborated by the results obtained by Girigoswami and co-workers [161]. The authors evaluated the effect of two liposomal formulations of xanthene dye Rhodamine 6G (R6G) (Figure 33) on a multidrug resistant *P. aeruginosa* present in a sewage water treatment plant. The liposomes were prepared from phospholipids extracted from hen eggs with the further addition of cholesterol. In one of the liposomal formulations, the authors used polyvinyl alcohol (PR6G) to stabilize the liposome thin films (obtained by the hydration method) and to minimize reticuloendothelial system uptake. The other positively charged formulation (CR6G) was obtained by covering the films with the cationic quaternary surfactant amine hexadecyltrimethylammonium bromide (CTAB). The results showed that, in terms of ^1^O_2_ quantum yield, both formulations are more efficient generators than R6G alone, leading to a more effective photodynamic inactivation of *P. aeruginosa*. However, despite the cationic formulation being less efficient than PR6G in generating ^1^O_2_, it showed a faster decrease in bacterial survival than PR6G. The authors explained that the electrostatic interactions between the superficial positive charge in CR6G and the cell surface of this Gram-(−) bacterium were responsible by this higher efficiency [161].

Recent studies also show that the incorporation of the PS in liposomes can accelerate the practical application of photodynamic inactivation to endodontic infections as a new clinical protocol [162].

Azevedo and co-workers [163] evaluated the delivery and the photoxicity of aluminum chloride phthalocyanine (AlPc) (Figure 2) entrapped in cationic liposomes against cariogenic bacteria and pulp cells in caries lesions. The study showed that the AlPc-cationic liposome was preferentially absorbed by bacterial cells when compared to eukaryotic dental pulp cells, and the reduction of microbial load from bacterial cultures was evident. The clinical study with volunteers showed a mean reduction of 82% of total bacteria in the treated cavities after the PDT application.

**Figure 34:**
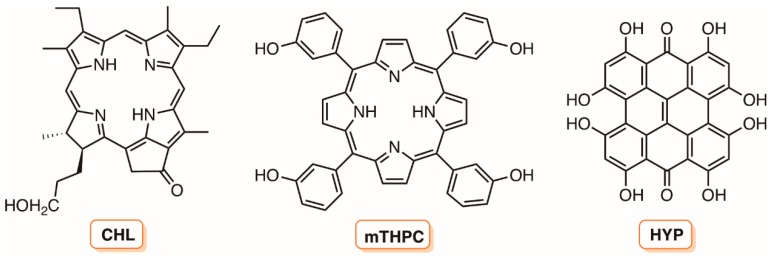
Structures of PS used after incorporation in liposomes formulations to efficiently photoinactivate bacteria and viruses.

Under the same context, the photodynamic efficacy of 5,10,15,20-tetrakis(3-hydroxyphenyl)chlorin (mTHPC) (Figure 34) in liposomes was investigated towards bacteria *E. faecalis* frequently found in persistent endodontic infections [164]. The results showed that the amount of mTHPC incorporated in the liposomal formulation formed by 9.1 mixture of dl-α-dipalmitoyl-phosphatidyl choline (DPPC) and dl-α-dipalmitoylphosphatidylglycerol (DPPG) (formulation Foslipos, Biolitec AG, Jena, Germany) and the light dose were important protocol features to consider during the photoinactivation treatment. *E. faecalis* was completely suppressed (reduction of > 8 log) after incubation with liposomes enriched with 50 µM mTHPC and illumination with 100 J cm^−2^. Irradiation of the non-photosensitized solution showed no suppressing impact and incubation of the PS without additional irradiation caused a maximal reduction of 1.5 log. The liposomal formulation Foslipos containing mTHPC was also able to completely eradicate *Streptococcus sobrinus* (reduction of 100% at 5 μg mL^−1^) and *S. mutants* (reduction of 100% at 1.25 μg mL^−1^) after 15 min of dark incubation and 2 min of blue light (400–505 nm) [165].

As an extension of the previous study, the German group evaluated the photodynamic killing of *E. faecalis* in dentinal tubules using the mTHPC incorporated in liposome formulation Foslipos (DPPC/DPPG) and in flexible invasomes containing soybean phosphatidyl, 10% ethanol, and 1% of a terpene mixture (d-limonene, citral and 1,8-cineole) [166]. The results showed that both mTHPC formulations had a significant antimicrobial effect and were able to suppress *E. faecalis* inside the dentinal tubules up to 300 μm. A bacterial reduction of up to 3.6 log mL^−1^ was ascertained for the mTHPC invasomes formulation directly at the root canal wall. It was also observed that photodynamic treatment using an invasomes formulation was even more effective than 1% chlorohexidine, a conventional disinfectant, which caused a bacterial reduction of only 1.2 log CFU. A very recent study concerning the limited use of hypericin (HYP, Figure 34) as a PS due to its highly lipophilic nature, poor solubility in aqueous media, and poor bioavailability showed that its incorporation in liposomes could be a promising alternative [167]. The results revealed that the liposomal formulations of DOPE/CHEMS/DPPC (1,2-dioleoyl-*sn*-glycero-3-phosphoethanolamine/cholesteryl hemisuccinate/l-α-dipalmitoylphosphatidylcholine), DSPC/DPPC/DSPE-PEG (1,2-distearoyl-*sn*-glycero-3-phosphatidylcholine/l-α-dipalmitoylphosphatidylcholine/1,2-distearoyl-*sn*-glycero-3-phosphoethanolamine-*N*-[methoxy(polyethylene glycol)-2000] and DPPC/DOTAP loaded with HYP were able to attain a 2.3–2.5 log reduction of *Staphylococcus saprophyticus* subsp. *bovis* after irradiation with a 589 nm diode laser at 22.2 J cm^−2^ and facilitated the binding and uptake of the PS through the bacterial cell wall.

Finally, considering that the viral safety of blood-derived products relies on properly chosen inactivation procedures, Sagristá et al. [168] reported a protocol based on an efficient incorporation of chlorin derivative (CHL) (Figure 34) into anionic unilamellar liposomes (POPC/OOPS) followed by its immobilization in the chromatographic support Sephacryl S-1000 beads. The photodynamic efficiency of the chlorin-containing liposomes formulation, free in solution and immobilized on the chromatographic support, was evaluated towards bovine viral diarrhea virus (BVDV) and encephalomyocarditis virus (EMCV). The results indicated that the enveloped virus BVDV could be successfully inactivated (>4 log) by both systems in culture medium but not the non-enveloped virus EMCV. The results suggested that the targets of the photodynamic action of chlorin CHL were the envelope components. The diminished effectiveness of CHL-containing liposomes in solution or immobilized in the chromatographic support when the culture media was replaced with human plasma was justified by the authors considering that plasma components hinder the access of the PS to the light.

## 6. Hydrogel Materials

The immobilization of PS in organic synthetic polymers can involve different approaches like the covalent or non-covalent coupling of the macrocycle with polymers with complimentary functionalities or the co-polymerization of PS with polymerizable moieties with monomers like styrene or methyl methacrylate [169,170,171,172,173]. Since in this review the preparation of some photoactive materials containing organic synthetic polymers was already referred, we decided in this section to give a special attention to photoactive materials based on hydrogels.

Hydrogels are three-dimensional networks composed of hydrophilic polymer chains. Many research groups are exploring them due to their efficacy in preserving the loaded cargo and due to their good responsiveness to the environment stimuli [174,175]. The hydrogel’s features make them good candidates for wound healing, implant/catheter coatings, skin infections treatment, and even orifice-barrier applications [176]. Therefore, several hydrogel-based systems have been selected in the last few years to overcome the low solubility of macrocycles, such as some porphyrin derivatives [177,178,179]. Their ability to encapsulate porphyrins by cross-linking them in the hydrogel structure avoids the leakage of the PS and also prevents their aggregation in aqueous media or physiologic conditions. In addition, the hydrogel carriers can commonly be designed to be biocompatible and/or biodegradable [180].

Thus, PSs immobilized on hydrogels are being considered to fight bacterial attachment onto intraocular lenses during cataract extraction and intraocular lens implantation, namely by bacteria such as *S. epidermidis*, which is the most frequently responsible for cases of acute endophthalmitis [181,182], and in some cases leading to the production of a microbial biofilm on the surface of the intraocular lenses [183]. Bearing this in mind, McGlinchey and co-workers characterized the physicochemical and the photoantimicrobial properties of hydrogel materials (anionic copolymers of 2-(hydroxyethyl)methacrylate with methacrylic acid), which have been surface-modified by impregnation with the tetracationic porphyrin TMPyP (Figure 2) [184]. This study enabled the discovery of candidate anti-infective hydrogel based intraocular lens materials to improve patient outcomes in cataract surgery. The anionic hydrogel copolymers were shown to permanently bind the cationic TMPyP by electrostatic interactions as a thin surface layer. The thermal and mechanical properties of the materials showed that the porphyrin acts as a surface cross-linking agent, yielding surfaces that were more hydrophilic. Thus, the microbiological studies showed that *S. epidermidis* adherence was reduced by up to 99.02 ± 0.42% relative to the control in intense light conditions, and 91.76 ± 5.99% in the dark. The ability to concentrate the photocytotoxic effect at a surface, together with a significant dark effect, was envisaged as a platform for a range of light-activated anti-infective biomaterial technologies. This was considered a great advantage since the treatment area can be strongly localized at the required point of bacterial contact with the intraocular lens surface. This can also avoid the side-effects associated with toxicity arising from accumulation of PS in the tissue [184].

Later, Gorman and colleagues also selected hydrogel materials (methyl methacrylate, methacrylic acid, 2-(hydroxyethyl)methacrylate, benzoyl peroxide, and ethylene glycol dimethacrylate), modified with the same tetracationic porphyrin (TMPyP), in order to get a hydrogel based intraocular lens materials. By doing this, the authors expected to obtain a material with antimicrobial properties, due to the photodynamic generation of ROS, and a material that was able to protect the retina from damaging of short wavelength visible light. The incorporation of the PS into hydrogels was accomplished by immersing samples of the materials in PBS solutions of TMPyP (2 min), followed by washing with deionized water for seven days. Then, the authors evaluated the resulting materials against both Gram-(+) *S. aureus* and Gram-(−) *P. aeruginosa*. At the end of the study, a significant reduction of bacterial adherence to the TMPyP-impregnated materials was observed when compared to untreated control materials, confirming the generation of ROS by the incorporated porphyrin upon photoactivation [185].

This type of hydrogel materials can also be used against bacterial biofilms. For instance, McCarron and co-workers [186] developed a poly(vinylalcohol)-borate hydrogel as a delivery system to achieve a photodynamic inactivation of wound infections. Therefore, they chose two cationic PSs (MB and TMPyP (Figure 2)) to be loaded into the hydrogel materials in order to photoinactivate an MRSA strain. After the biological assessment, they found that reductions of bacterial abundance in the biofilm was 88.19% for MB at a concentration of 10 μg mL^−1^ and a light dose of 100 J cm^−2^, while TMPyP (10 μg mL^−1^) caused a reduction of 99.71%. Despite the total bacterial biofilm eradication did not occur in both tested hydrogel materials and bacterial abundance was higher than 10^5^ CFU mL^−1^ in many cases, photodynamic inactivation of microorganisms was not associated with the development of resistance, even upon multiple treatments [186].

All these studies demonstrate that some of the hydrogel-based photoinactivation caused admirable antibacterial performance against both Gram-(−) and Gram-(+) bacteria in planktonic and biofilm form. Additionally, this type of treatment can be performed several times at one site due to the low fluidity of the hydrogels [187].

## 7. Supports Based on Carbon Nanomaterials

**Figure 35:**
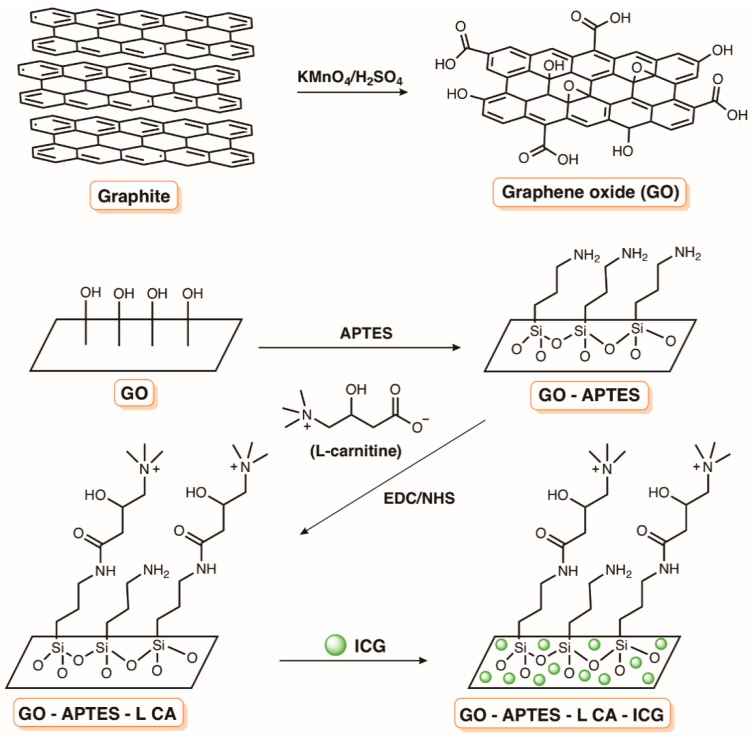
Functionalization of GO with APTS and L CA and incorporation of ICG (adapted from Reference [189]).

The unique properties of graphene oxide, such as a large surface area decorated with different functional groups, biocompatibility, a lack of obvious toxicity, photothermal activity, intrinsic high near-infrared absorbance, and water solubility, are making it an ideal material to carry PSs [188]. Under this context, and considering the attention given to PDI as an effective approach to improve root canal disinfection, Tayebeh Akbaria et al. [189] used functionalized nano-graphene oxide (GO) with APTS and then with l-carnitine (GO-APTES-L CA) as a platform to load, via π-π stacking interactions, the amphiphilic dye indocyanine green (ICG), also known as Cardio-Green^®^ (Figure 35). This dye, with a unique peak absorbance at 780 nm, has limitations such as little photostability and concentration-related aggregation. The success of the incorporation was confirmed by adequate techniques (FT-IR, SEM and UV–Vis spectrometry) and the composite GO-APTES-L CA-ICG obtained contained 200 μg mL^−1^ of ICG. The antimicrobial and anti-biofilm potential of GO-APTES-L CA-ICG was assessed against *E. faecalis*, both in planktonic and biofilm forms. PDI mediated by GO-APTES-L CA-ICG at an energy density of 31.2 J cm^−2^ showed a significant reduction (2.81 log) in the count *of E. faecalis* and a reduction of up to 99.4% on the biofilm formation ability. The overall antimicrobial and anti-biofilm potential of GO-APTES-L CA-ICG was higher than PDI based on ICG (1000 μg mL^−1^) (47% and 21%, respectively). The authors verified that the composite GO-APTES-L CA-ICG with a higher incorporation of ICG (400 μg mL^−1^) after a total light dose irradiation of 31.2 J∙cm^−2^ showed a significantly greater reduction in the number of *E. faecalis* than PDI based on ICG alone (1000 μg mL^−1^) [190].

**Figure 36:**
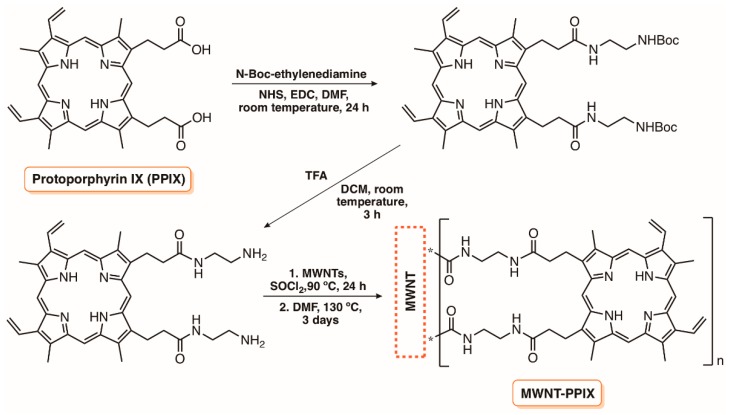
Functionalization of MWNT with PPIX (adapted from Reference [191]).

Considering the attractive properties of carbon nanotubes to develop ultrathin, flexible, and transparent films for immobilization, accompanied by their ability to photogenerate ROS, Kane and co-workers [191] reasoned that their efficiency could be significantly enhanced by functionalization of the nanotubes with porphyrins due to their high efficiency in generating ROS. The authors developed nanocomposite films based on the conjugation of multiwalled carbon nanotubes (MWNT) and protoporphyrin-IX (PPIX) (Figure 36) and evaluated their photoinactivation efficiency towards *S. aureus*. The coupling involved the previous functionalization of the acid groups of protoporphyrin-IX with amino groups followed by their condensation with the acid groups of MWNT after their activation with SOCl_2_. The biological results showed that after 1 h of visible light irradiation, the nanocomposite films reduced 15–20% of *S. aureus* survival on plate surface. As an extension of the previous studies, the authors tested the MWNT-PPIX ability to inactivate the Influenza A virus (Figure 36) [192]. The strain used was Influenza A/X-31, which is a high-yield H3N2 strain, commercially available and developed for designing vaccines. The conjugate was found to be highly active in reducing the ability of the influenza virus to infect mammalian cells, and the study showed that the conjugate can be recovered from an aqueous solution and reused at least three times without any significant loss of its activity. Therefore, it was proposed that in addition to being effective bactericidal agents, the MWNT-PPIX conjugates may be applied as reusable antivirals in wastewater treatment, as they can be easily recovered without leaving any toxic by-products.

**Figure 37:**
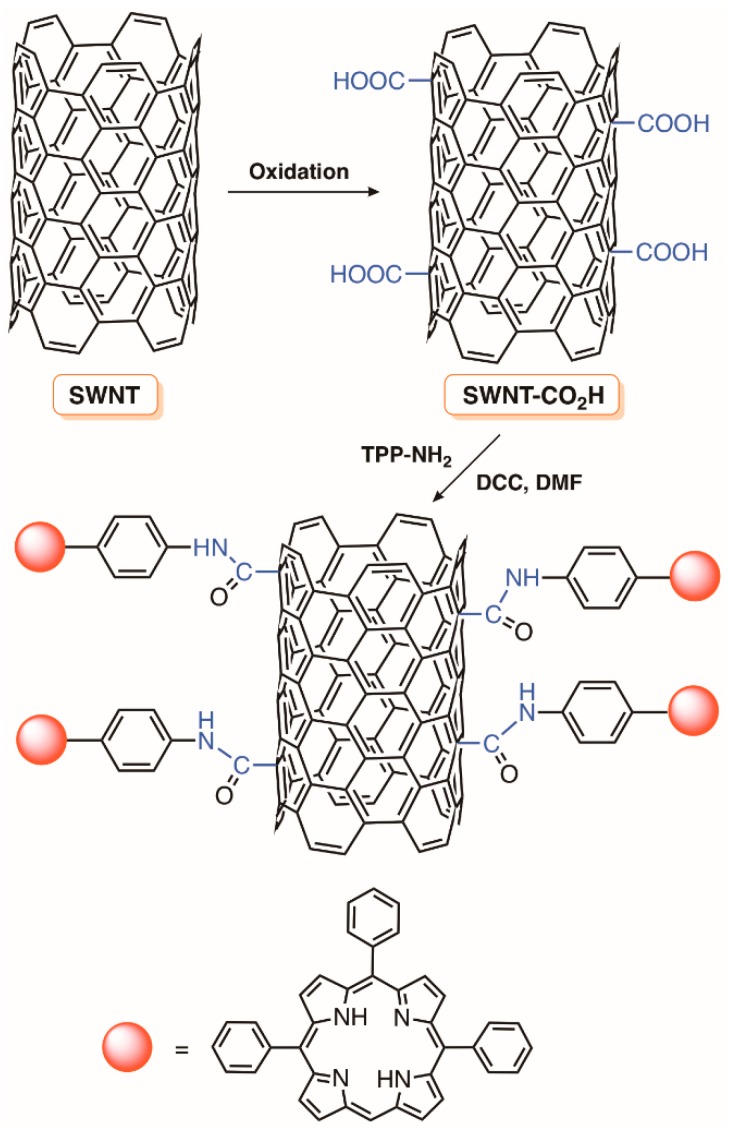
Preparation of SWNT and functionalization with TPP-NH_2_ (adapted from Reference [193]).

Recently, Gopinath and co-workers [193] also developed a nanocomposite made up of single-walled carbon nanotubes (SWCNTs) covalently linked to the amine-functionalized porphyrin 5-(4-aminophenyl)-10,15,20-triphenylporphyrin (TPP-NH_2_) and evaluated its antimicrobial potential after activation with visible light (Figure 37). The results showed that the use of an appropriate amount of single-walled carbon nanotubes (SWCNTs) led to cell membrane damage. Consequently, the SWCNT-porphyrin conjugates can be used as an antibacterial agent.

## 8. Final Remarks

PDI appears as an effective method to inactivate a broad spectrum of planktonic and biofilm embedded microorganisms. In addition, the development of photoactive materials has provided an opportunity to improve several aspects of treatment, namely the possibility to increase the efficiency of the photodynamic treatment without inducing the resistance mechanism of microorganisms. Additionally, this type of technology offers the possibility to recover and reuse the photosensitizing system in an eco-friendly manner. Although, the application of photoactive materials in the field of PDI solved some difficulties of this therapy, there is still room for new improvements. It is expected that the information compiled in this review can motivate the development of other practical and efficient approaches for PDI in order to respond to its currently underutilization in clinic and environmental applications.
